# The multicellular signalling network of ovarian cancer metastases

**DOI:** 10.1002/ctm2.633

**Published:** 2021-11-08

**Authors:** Leah Sommerfeld, Florian Finkernagel, Julia M. Jansen, Uwe Wagner, Andrea Nist, Thorsten Stiewe, Sabine Müller‐Brüsselbach, Anna M. Sokol, Johannes Graumann, Silke Reinartz, Rolf Müller

**Affiliations:** ^1^ Department of Translational Oncology, Center for Tumor Biology and Immunology (ZTI) Philipps University Marburg Germany; ^2^ Clinic for Gynecology, Gynecological Oncology and Gynecological Endocrinology University Hospital (UKGM) Marburg Germany; ^3^ Genomics Core Facility, Center for Tumor Biology and Immunology (ZTI) Philipps University Marburg Germany; ^4^ Institute of Molecular Oncology Philipps University Marburg Germany; ^5^ The German Centre for Cardiovascular Research (DZHK), Partner Site Rhine‐Main Max Planck Institute for Heart and Lung Research Bad Nauheim Germany; ^6^ Institute for Translational Proteomics, Philipps University Marburg Germany

**Keywords:** adipocyte, carcinoma‐associated fibroblast, HSP70, mesothelial cell, metastasis, omentum, ovarian carcinoma, signaling network, tumour microenvironment, WNT4

## Abstract

**Background:**

Transcoelomic spread is the major route of metastasis of ovarian high‐grade serous carcinoma (HGSC) with the omentum as the major metastatic site. Its unique tumour microenvironment with its large populations of adipocytes, mesothelial cells and immune cells establishes an intercellular signaling network that is instrumental for metastatic growth yet poorly understood.

**Methods:**

Based on transcriptomic analysis of tumour cells, tumour‐associated immune and stroma cells we defined intercellular signaling pathways for 284 cytokines and growth factors and their cognate receptors after bioinformatic adjustment for contaminating cell types. The significance of individual components of this network was validated by analysing clinical correlations and potentially pro‐metastatic functions, including tumour cell migration, pro‐inflammatory signal transduction and TAM expansion.

**Results:**

The data show an unexpected prominent role of host cells, and in particular of omental adipocytes, mesothelial cells and fibroblasts (CAF), in sustaining this signaling network. These cells, rather than tumour cells, are the major source of most cytokines and growth factors in the omental microenvironment (*n* = 176 vs. *n* = 13). Many of these factors target tumour cells, are linked to metastasis and are associated with a short survival. Likewise, tumour stroma cells play a major role in extracellular‐matrix‐triggered signaling. We have verified the functional significance of our observations for three exemplary instances. We show that the omental microenvironment (i) stimulates tumour cell migration and adhesion via WNT4 which is highly expressed by CAF; (ii) induces pro‐tumourigenic TAM proliferation in conjunction with high CSF1 expression by omental stroma cells and (iii) triggers pro‐inflammatory signaling, at least in part via a HSP70–NF‐κB pathway.

**Conclusions:**

The intercellular signaling network of omental metastases is majorly dependent on factors secreted by immune and stroma cells to provide an environment that supports ovarian HGSC progression. Clinically relevant pathways within this network represent novel options for therapeutic intervention.

## Graphical abstract

Besides macrophages, omental adipocytes, mesothelial cells and fibroblasts play an unexpectedly prominent role in the pro‐metastatic signaling network of ovarian carcinoma, encompassing clinically relevant pathways and potential therapeutic targets. The central role of stroma‐derived mediators is exemplified by (i) WNT4 stimulating tumour cell adhesion and migration, (ii) activation of pro‐inflammatory signaling by extracellular HSP70 and (iii) CSF1 inducing macrophage proliferation.

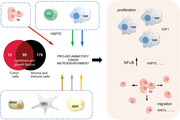

## 1 INTRODUCTION

High‐grade serous carcinoma (HGSC) is the most frequent and fatal of all gynaecologic cancers, mainly due to its early and widespread transcoelomic dissemination to peritoneal surfaces in abdominal and pelvic cavities. Transcoelomic spread is enabled by the peritoneal fluid which provides a carrier for tumour cells that are shed from solid tumour lesions.^1^ At advanced stages of HGSC the peritoneal fluid increases to large volumes, referred to as malignancy‐associated ascites. Ascites not only serves as a passive carrier, but also provides a tumour‐promoting and immune suppressive environment mediated by soluble mediators as well as extracellular microvesicles.^2^, ^3^ Due to its high content in bioactive compounds and its active role in peritoneal dissemination, HGSC ascites functionally differs from other human cancers, where effusions are usually reactive or represent an epiphenomenon.

The most frequent metastatic site for HGSC is the omentum,^4^ a specialised adipose tissue connected by a mesothelial layer to other intraperitoneal organs.^5^ A specific feature of the omentum are regions referred to as milky spots, which mainly consist of macrophages and lymphocytes, which contribute to peritoneal immune surveillance.^5^, ^6^, ^7^ Besides immune cells other cell types of the omentum have been reported to promote ovarian cancer growth and metastatic spread. Thus, hypoxic mesothelial cells (MESO) at milky spots secrete vascular endothelial growth factor (VEGFA), thereby potentially inducing neo‐angiogenesis.^8^ Another example are omental adipocytes (ADI), which promote the homing and invasion of ovarian cancer by multiple mechanisms, including the secretion of adipokines, the promotion of tumour cell metabolism through the direct transfer of lipids to cancer cells^9^, ^10^, ^11^ and the ADI‐induced phosphorylation of salt‐inducible kinase 2 (SIK2), mediating AMPK‐dependent acetyl‐CoA carboxylase phosphorylation and PI3K/AKT activation in tumour cells.^12^ Furthermore, cancer‐associated fibroblasts (CAF), derived from omental fibroblasts and mesenchymal stem cells under the influence of the tumour microenvironment,^9^, ^13^ produce numerous factors imping on cancer cells to promote glycolytic metabolism, proliferation, invasion, angiogenesis and metastatic colonisation.^9^, ^14^, ^15^

Besides these omental cell types, tumour‐associated macrophages (TAM) clearly are instrumental in metastasis, in particular the CD163^+^Tim4^+^ subset, which promotes the stemness, invasive properties and epithelial to mesenchymal transition (EMT) of tumour cells by paracrine mechanisms.^16^ While omentectomy or depletion of CD163^+^Tim4^+^ cells prevented metastatic dissemination in a mouse model of ovarian cancer, depletion of monocyte‐derived TAM had little impact, emphasising the role of resident omental macrophages. In keeping with these findings, TAM from HGSC patients show a high degree of phenotypic, ontogenetic and tumour‐promoting heterogeneity, reflected, for example, by the differential expression of CD163 and cytokines associated with tumour progression.^17^, ^18^

Recent progress in single‐cell RNA sequencing (scRNA‐Seq) confirmed the intra‐ and inter‐patient heterogeneity of TAM in HGSC ascites.^19^ The authors identified four clusters characterised by genes associated with divergent phenotypes and functions, including immune stimulation (*HLA* genes, *IFNGR1, CD1D*), complement factor components and cathepsins, and markers of M1 and M2 differentiation. Likewise, CAF from ascites were similarly heterogeneous with four clusters defined by immune‐related genes, complement factors, chemokines (*CXCL1/2/10/12)* and cytokines (*IL6, IL10*). Previous work had identified four subtypes of HGSC, that is differentiated, proliferative, mesenchymal and immunoreactive, of which the latter two are linked to a poor or favourable outcome, respectively.^20^, ^21^ Intriguingly, scRNA‐Seq^19^ showed weak or no expression of mesenchymal and immunoreactive signatures by HGSC cells, but high expression by CAF and TAM clusters, respectively. This strongly suggests that the mesenchymal and immunoreactive subtypes are defined by the abundance of CAF and TAM rather than by cancer cell subpopulations, providing further evidence for the crucial role of tumour‐associated host cells. Interestingly, the fraction of TAM and CAF in tumour tissue appears to increase during progression, as suggested by a previous scRNA‐Seq study.^22^ Other scRNA‐Seq studies also confirmed a substantial heterogeneity among tumour cells^23^, ^24^ and lead to the proposal that HGSC is defined by continuous tumour evolution with mixtures of subclones and stage‐dependent infiltration of host cells rather than by discrete transcriptome subtypes.^23^

Despite considerable progress over the past years, our knowledge of the intercellular signalling network operating in HGSC metastases remains fragmentary. Published systematic transcriptomic studies suitable for the development of signalling networks are limited to ascites cells,^17^, ^25^ and were partly performed by scRNA‐Seq,^19^, ^22^, ^23^, ^26^ which is not informative for a subset of weakly expressed genes and possesses a limited power for differential expression studies compared to bulk analyses.^27^, ^28^, ^29^, ^30^, ^31^, ^32^ It is also currently unclear, to which extent ascites‐derived cell types resemble their counterparts in solid tumour lesions, since unbiased omics analysis have not been described for tumour‐associated non‐immune cells from HGSC patients.

**FIGURE 1:**
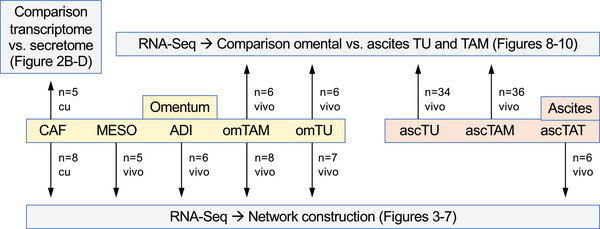
Workflow and conception of the present study. *n*: number of independent samples (different patients); vivo: samples directly used after isolation from clinical material; cu: cells cultured for xxx passages after isolation from omental metastases (CAF only). RNA‐Seq samples with contaminating samples of >6% were excluded from further analyses and the remaining samples were adjusted for minor contaminations by a bioinformatic approach

In the present study, we have performed systematic transcriptomic bulk analyses of all major cell types from omental HGSC metastases, supported and extended by proteomic and functional studies to (i) construct a comprehensive network of cytokines, growth factors and ECM components and their cognate receptors and (ii) compare the expression of these proteins in omental versus ascites‐derived TU and TAM. The workflow and general strategy of our study in schematically summarised in Figure 1. We will refer to TAM and TAT collectively as tumour‐associated ‘immune cells’ and to the compartment of ADI, MESO and fibroblasts (CAF) as tumour‐associated ‘stroma cells’ throughout this manuscript.

## 2 METHODS

### 2.1 Patient samples

Ascites and greater omentum tissue with metastatic lesions were collected from patients with ovarian HGSC undergoing primary surgery at the University Hospital in Marburg. Patient characteristics are summarised in Table S1. Clinical courses were evaluated by RECIST criteria^33^ in patients with measurable disease or profiles of serum CA125 levels according to the recommendations by the Gynecologic Cancer InterGroup (GCIG). Only patients with observations periods ≥12 months after first‐line surgery were included in the survival analysis.

### 2.2 Isolation of cells from HGSC ascites

Tumour cell spheroids, TAM and TAT were isolated from ascites essentially as described.^17^, ^25^ Briefly, mononuclear cells were isolated from ascites by density gradient centrifugation (Lymphocyte Separation Medium 1077; PromoCell). Medium size (‘M’) and large (‘L’) tumour cell spheroids were obtained by filtration using 30 and 100 μm cell strainer (Miltenyi Biotech). Smaller tumour spheroids (<30 μm; ‘s’) and single tumour cells (sc) were further enriched by depletion of CD45^+^ leukocytes by magnetic cell sorting (MACS; Miltenyi Biotech, Bergisch Gladbach, Germany). TAM were purified by selection for CD14^+^ cells on MACS microbeads. TAT were isolated from ascites as CD3^+^ cells by MACS. All microbeads for MACS (CD3, CD14, CD45) were obtained from Miltenyi Biotech. Cell populations with a purity of > 95%, as determined by flow cytometry, were used for subsequent analysis.

### 2.3 Isolation of tumour and host cells from omentum

Prior to enzymatic digestion, omentum tissue without visible metastatic lesions was dissected from omentum tumour tissue. ADI were obtained from omental tissue by digestion with 370 U/ml collagenase (Sigma Aldrich) in 10 ml adipocyte digestion buffer (5 mM D‐Glucose, 1.5% BSA in PBS) per 5 g tissue for 1 h at 37°C under continuous shaking. The disaggregated cell suspension was filtered through a 400 μm filter and centrifuged (5 min, 150× *g*) to separate ADI by density. The supernatant containing ADI was gently washed twice with PBS. During the washing steps, contaminating cells were separated from the floating ADI layer by gravity for 5 min. This process normally yields ADI fractions with a purity of >95% which were directly used for secretome cultures or lysed in PeqGold TriFastTM (Peqlab, Erlangen, Germany) for RNA isolation. Contamination of ADI with leukocytes or omTU was determined by fluorescence microscopy using FITC‐labelled anti‐human CD45 and PE‐labelled anti‐human EpCAM (both Miltenyi) combined with Hoechst 33342 staining (Thermo Fisher Scientific, Schwerte, Germany).

For the isolation of MESO, omental tissue was washed with PBS, minced into small pieces (approx. 5 mm diameter) and subsequently digested with trypsin (20 ml 0.05% Trypsin/0.02% EDTA per 10 g tissue) for 30 min at 37°C. The digested tissue was passed through a 100 μm nylon filter and centrifuged (10 min at 300 g). This MESO‐enriched fraction was cryopreserved for later purification my MACS.

To isolate tumour cells, CAF and omTAM, omental tumour tissue was treated as described for MESO, except that trypsin digestion was performed for 2 h at 37°C. The dissociated cell suspension was cryopreserved for later purification of omTU and omTAM by MACS. The residual tissue was consecutively treated with a mixture of 18.5 U/ml collagenase and 2.5 μg/ml hyaluronidase (Sigma Aldrich, Taufkirchen, Germany) in 20 ml fibroblast culture medium [DMEM/HAMs F12 (1:1), 10% FCS, 10 ng/ml EGF, 1% Pen/Strep] overnight at 37°C, filtered through a 100 μm mesh and washed as described above to yield a cellular fraction enriched for tumour cells, omTAM and CAF for cryopreservation.

The isolation of omTU, omTAM, MESO and CAF was achieved by different MACS sorting strategies. Cryopreserved cellular fractions after enzymatic digestion were thawed and processed over a Ficoll gradient to eliminate dead cells. omTUs were purified from tumour‐enriched fractions (after overnight digestion with trypsin, collagenase and hyaluronidase as described above) by MACS depletion of CD45^+^ leucocytes in combination with positive MACS selection for EpCAM^+^ cells (Miltenyi Biotech). CD14^+^ microbeads were used to isolate omTAM from the tumour fractions (after 2‐h digestion with trypsin, collagenase and hyaluronidase as described above) by positive MACS selection. CAF were obtained from the collagenase/hyaluronidase fraction (described above) after initial preculture in fibroblast medium [DMEM/Ham's F12 (1:1) + 10% FCS + 1 ng/ml EGF + 1% penicillin/streptomycin] and subsequent MACS depletion of CD45^+^ leucocytes and EpCAM^+^ tumour cells. If necessary, a positive selection using anti‐fibroblast beads (Miltenyi Biotech) was additionally applied to yield sufficiently pure CAF. The same purification strategy using CD45 and EpCAM depletion was used in parallel to isolate MESO from the 30 min trypsin digest fraction obtained from macroscopic tumour‐free omentum tissue.

RNA was isolated from all cell types directly after purification (ex vivo) except for CAF, which were cultured in OCMI medium^34^ supplemented with 50% ascites for a maximum of three passages.

### 2.4 Flow cytometry analyses

Cells isolated from ascites or omentum were analysed by flow cytometry performed on a FACS Canto II instrument using Diva Software (BD Biosciences, Heidelberg, Germany) using the following staining protocol. Tumour cells were identified with Vioblue‐labelled anti‐human EpCAM (Miltenyi Biotech), TAM with FITC‐labelled anti‐human CD14 (Miltenyi Biotech) and TAT with APC‐labelled anti‐human CD3 (Biolegend, Koblenz, Germany). MESO and CAF were characterised by negative staining with Vioblue‐labelled anti‐EpCAM and further discriminated using fibroblast markers like PE‐labelled anti‐human CD140a (eBioscience/Thermo Fisher Scientific) and FAP (R&D Systems//Thermo Fisher Scientific) in combination with APC‐labelled anti‐human mesothelin (R&D Systems) and intracellular staining with APC‐labelled anti‐human cytokeratin and FITC‐labelled anti‐human vimentin (both from Miltenyi). In some cases, anti‐human MUC16 antibody (clone OC125, Sigma Aldrich) combined with secondary FITC‐labelled anti‐mouse IgG (eBioscience) was included. Isotype control antibodies were purchased from BD Biosciences, Miltenyi Biotech and eBioscience. Results were calculated as percentage of positive cells and mean fluorescence intensities (MFI). Cell death was assessed by propidium iodide staining. Proliferation of TAM was analysed by staining with anti‐human CD14 FITC (Miltenyi) and intracellular staining with anti‐Ki67 APC (Biolegend).

### 2.5 Protein mass spectrometry (MS) of conditioned media from CAF

**FIGURE 2:**
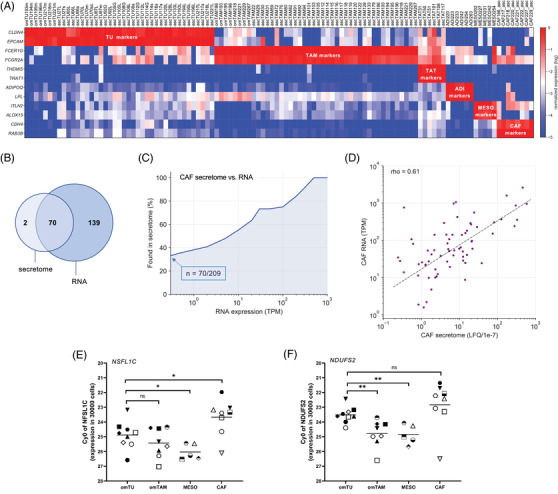
Validity of RNA‐Seq data. (A) Heatmap depicting relative cell type‐specific marker gene expression (TPM) in samples isolated from HGSC ascites and omentum after bioinformatic adjustment for contaminating cells. Samples with >6% of any contaminating cell type were excluded prior to adjustment. TPM values were gene‐wise normalised across all samples (highest expression level = 10^0^). Each datapoint represents an independent sample. The corresponding non‐adjusted data is shown in Figure S1. See Figure S1 for further details. (B) Venn diagram depicting the number of cytokines and growth factors identified in the CAF secretome and transcriptome (TPM > 0.3). (C) Presence of cytokines and growth factors in the CAF secretome (*n* = 5; determined by MS‐based proteomics of CM) in relation to the level of RNA expression (TPM). Details are shown in Table S9. (D) Correlation of signals obtained by RNA‐SEquation (median TPM) and MS‐based proteomics (median LFQ) for all proteins found in the CAF secretome. (E, F) Assessment of cell‐type‐dependent RNA content. RNA from equal numbers of omTU, omTAM and CAF (*n* = 30 000 cells) was analysed using primers for *NDUFS2* and *NFSL1C* mRNA without normalisation. *NDUFS2* and *NFSL1C* were chosen due to a very low variance across all samples and cell types. Each symbol indicates an individual patient. If possible, samples of different cell types were matched (i.e. from the same patient). *p < .05; **p < .01; ns, not significant by unpaired *t*‐test

For proteomic analyses of conditioned media, omental CAF or ascTU were first propagated in OCMI medium with 50% pooled ascites. After 16 h at 37°C and 5% CO_2_, the cells were washed three times in PBS and twice in serum‐free medium M199 (Gibco, Thermo Fisher Scientific) mixed with an equal volume of DMEM/Ham's F‐12 (Biochrom, Schaffhausen, Germany) and cultured in serum‐free medium for another 0 or 20 h before harvesting the supernatants for MS‐based proteomic analysis. Following acetone precipitation from supernatants, up to 40 μg of proteins were loaded on a gradient gel (NuPAGE 4–12% Bis‐Tris gel, Invitrogen) and separated by SDS‐PAGE prior to in‐gel digestion.^31^ Analysis by liquid chromatography/tandem mass spectrometry (LC/MS2) was performed as described^17^ and peptide/spectrum matching as well as label free quantitation used the MaxQuant suite of algorithms^35^, ^36^, ^37^ against the human Uniprot database (canonical and isoforms; downloaded on 2020/02/05; 1888349 entries). Relevant instrument parameters were extracted and summarised using MARMoSET (see Supporting Information MS settings). Protein‐specific signals were calculated by subtracting the 0‐h LFQ value from the 16‐h LFQ log10 value. Data in Figures 2D and 8D are represented as LFQ/10.^7^ For Figure 1C, proteins were considered ‘in secretome’ if there was a peptide group (identified in any sample by MaxQuant) associated to them and a higher median signal in 20‐h samples compared to 0 h.

### 2.6 Transient WNT4 knockdown in CAF by siRNA transfection

siRNA transfection was performed in CAF from omental metastasis cultured in OCMI plus 5% FCS using the TransIT‐X2 reagent from Mirus (Madison, WI, USA) or lipofectamine 3000 (Invitrogen, Thermo Fisher Scientific), according to the manufacturer's protocol. siWNT4 ON‐Target plus smartpool from Dharmacon (Horizon Discovery, Cambridge, UK) and MISSION siRNA Universal Negative Control # 2 (Sigma Aldrich) as a control siRNA were used. Untransfected CAF were included as controls. Cells were harvested 48 h after transfection for RNA and protein expression analyses and for generation of conditioned media.

### 2.7 Transient WNT4 overexpression in LP9 cells

The human MESO line LP9 (AG07086, Coriell Institute, Camden, NJ) with low basal WNT4 expression level was chosen to induce WNT4 overexpression by transient transfection with WNT4_pCDNA3.1 vector (WNT4_OE) or empty pCDNA3.1 control (pCDNA3.1_Ctrl) (GenScript Biotech, Piscataway, NJ) using Lipofectamine 3000 (Invitrogen, Thermo Fisher Scientific) according to the manufacturer's instructions. WNT4 expression was validated 48 h after transfection by RT‐qPCR (primers in Table S2). WNT4 secretion was determined in conditioned media by Western Blot analysis.

### 2.8 Tumour cell migration

The impact of WNT4 secretion on tumour migration was evaluated in two Transwell assay formats using the WNT4^low^ ovarian cancer cell line OVCAR‐4 (NIGMS Human Genetic Cell Repository of the NIH), which express the main WNT4 receptor FZD8 and coreceptors LRP5/6. In the first setting, tumour migration was determined in the presence of conditioned medium (CM) from LP9 cells transiently transfected with WNT4_pCDNA3.1 for overexpression of WNT4 as chemoattractant. CM from LP9 cells transfected with empty vector pCDNA3.1 and untransfected LP9 cells were included as controls. Briefly, 50 000 OVCAR‐4 cells were seeded in 300 μl serum‐free RPMI 1640 medium (Life Technologies, Darmstadt, Germany) per transwell insert (8.0 μm pore size; BD Biosciences). CM from LP9 cells (1:4 diluted in serum‐free medium) or 10% FCS as positive control were added as chemoattractant to the lower chamber. The cells were allowed to migrate through the filter for 28 h at 37°C in a 5% CO_2_ incubator. Filters were stained with crystal violet (0.2% in 20% methanol, 1:5 dilution) for 10 min and evaluated under a Leica DMI3000B microscope (Leica, Wetzlar, Germany). Migrated cells were counted in >7 visual fields per filter using the ImageJ software. In an alternative approach, OVCAR‐4 cells or CellTracker green CMFAD‐labelled primary ascites‐derived tumour cells (ascTu OC_280, OC_261, see Section 2.10) were pre‐incubated with 1:3 diluted CM of LP9 cells transiently overexpressing WNT4 or control CM (see above) for 24 h at 37°C and 5% CO_2_ prior to performing transwell migration assays with 10% FCS as chemoattractant as described above.

### 2.9 Wound healing assay

Forty thousand OVCAR‐4 cells were grown in culture chambers with 4‐well silicone inserts (Cat# 80469; IBIDI, Gräfelfing, Germany) for 24 h. After serum starvation in RPMI1680 with 1% FCS for 24 h, cells were treated with conditioned media (1:3 diluted) from transiently transfected LP9 cells (WNT4_pCDNA3.1 or empty vector pCDNA3.1) or from untransfected LP9 cells for additional 24 h. Silicon Inserts were removed and gap closure by cell migration was monitored by microscopy at time points 0, 8 and 24 h and analysed using ImageJ software.

### 2.10 Tumour cell attachment to mesothelial cells

Omentum‐derived MESO (OC_140; OC_280) were plated in collagen‐I‐coated (5 μg/cm^2^; Gibco/Thermo Fisher Scientific) 96‐well plates (25 000 cells/well) and grown to confluency in OCMI/5% FCS for 3 days at 37°C, 5% CO_2_. The integrity of the MESO layer was evaluated by microscopy (Figure S11C). OVCAR‐4 cells or primary ascites‐derived tumour cells (ascTu OC_280) were pre‐incubated with 1:3 diluted CM of LP9 cells transiently overexpressing WNT4 or control CM (see above) for 24 h. Tumour cells were harvested, labelled with 10 μM CellTracker green CMFDA (Invitrogen/Thermo Fisher Scientific) for 30 min and washed with OCMI/5%FCS. Five thousand labelled tumour cells were added to MESO monolayers for 1 h (OVCAR4) or 2 h (ascTU), at 37°C. Plates were washed and attached tumour cells were evaluated under a Leica DMI3000B fluorescence microscope (Leica, Wetzlar, Germany). Cells were counted in 9 visual fields per preparation (3 × 96 wells per preparation were evaluated) using the ImageJ software.

### 2.11 Treatment of TAM with rhHSP70

Cryopreserved primary ascTAM derived from different ovarian cancer patients were cultured in ascites (pool of 10 different patients) for 6 days followed by overnight starvation in RPMI1680 medium supplemented with 1 mM sodium pyruvate (Sigma Aldrich). Cells were treated with 1 μg/ml recombinant human Hsp70 protein (rhHsp70, low endotoxin; Enzo Life Sciences, Lörrach, Germany). A control (Ctrl^low^) containing 0.005 ng/ml LPS from *E. coli* (Sigma Aldrich) corresponding to the endotoxin level of the rhHSP70 (indicated by the manufacturer) was included. To further address the potential effect of endotoxin contaminations of rhHSP70, TAM were pre‐incubated with 10 μg/ml polymyxin B (PMB, Sigma Aldrich) for 2 h prior to stimulation.

### 2.12 IL‐6 quantification by ELISA

IL‐6 in culture supernatants of ascTAM after stimulation with rhHSP70 in the presence and absence of Polymyxin B (Sigma‐Aldrich) was quantified by ELISA (Invitrogen/Thermo Fisher Scientific) according to the manufacturer´s instructions.

### 2.13 Immunoblotting and quantification

Immunoblots were performed according to standard western blotting protocols using the following antibodies: α‐p65 monoclonal antibody (Cell Signaling Technology, Frankfurt, Germany; Cat# 8242, RRID:AB_10859369); α‐GAPDH polyclonal antibody (Sigma‐Aldrich; Cat# G9545, RRID:AB_796208); α‐WNT4 (Clone 55025) monoclonal antibody (R&D Systems; Cat# MAB4751, RRID:AB_2215448); α‐Lamin B1 polyclonal antibody (MyBioSource, San Diego, CA; Cat# MBS422963); α‐IKKalpha/beta (H‐470) polyclonal antibody (Santa Cruz Biotechnology, Heidelberg, Germany; Cat# sc‐7607, RRID:AB_675667); α‐rat IgG horseradish peroxidase (HRP)‐linked AB (R&D Systems; Cat# HAF005, RRID:AB_1512258), α‐goat IgG HRP‐linked polyclonal antibody (Jackson ImmunoResearch Labs/Dianova, Hamburg, Germany; Cat# 705‐035‐003, RRID:AB_2340390), α‐rabbit IgG HRP‐linked polyclonal antibody (Cell Signaling Technology; Cat# 7074, RRID:AB_2099233) and α‐mouse IgG HRP‐linked polyclonal antibody (Cell Signaling Technology; Cat# 7076, RRID:AB_330924). For validation of WNT4 secretion, conditioned media of transiently transfected LP9 cells were concentrated 10‐fold using a vacuum concentrator. To determine HSP70‐dependent nuclear translocation of p65, subcellular fractionation of stimulated TAM was performed as previously described.^38^ Imaging and quantification were carried out using the ChemiDoc MP system and Image Lab software version 5 (Bio‐Rad, Feldkirchen, Germany). The signals of the phosphorylated forms were normalised against the respective protein signals.

### 2.14 RT‐qPCR

cDNA isolation and qPCR analyses were performed as described,^25^, ^39^ using *RPL27* for normalisation, except for *NDUFS2* and *NFSL1C* which were analysed without normalisation (Figure 1E and F). Raw data were evaluated by the Cy0 method.^40^ Primer sequences are listed in Table S2.

### 2.15 RNA‐Seq

RNA‐Seq was carried out on an Illumina NextSeq 550 as described.^41^ Data were aligned to the human genome retrieve from Ensembl using STAR (version STAR_2.6.1d).^42^ Gene read counts were established as read count within merged exons of protein coding transcripts (for genes with a protein gene product) or within merged exons of all transcripts (for non‐coding genes) and normalised to TPM (transcripts per million) or CPM (counts per million) as appropriate for the library type (see below). TPM were calculated based on the total exon read counts and length of merged exons. All genomic sequence and gene annotation data were retrieved from Ensembl release 96, genome assembly hg38.

RNA quality was assessed using the Experion RNA StdSens Analysis Kit (Bio‐Rad). Only samples with a RNA quality index ≥8.0 were included in this study. RNA‐Seq libraries were constructed using ‘Illumina Truseq Stranded total RNA’ (Illumina, Berlin, Germany) for ascites cells (previously published datasets E‐MTAB‐3167, E‐MTAB‐4162, E‐MTAB‐5199, E‐MTAB‐5498), ‘Illumina Truseq Stranded mRNA’ for ascites and omentum cells (deposited as E‐MTAB‐10611 at EBI ArrayExpress) and ‘Lexogen Quantseq 3′mRNA‐seq Libarry Prep Kit FWD for Illumina’ (Lexogen, Vienna, Austria) in combination with the ‘Lexogen UMI Second Strand Synthesis Module for QuantSeq FWD (Illumina, Read 1)’ for CAFs (E‐MTAB‐10611), according to the manufacturer's instructions. Quality of sequencing libraries was controlled on a Bioanalyzer 2100 using the Agilent High Sensitivity DNA Kit (Agilent, Waldbronn, Germany). Pooled sequencing libraries were quantified and sequenced on the Illumina NextSeq550 platform with 75 base single reads.

To test the possibility to use these datasets in comparative analyses, we determined the impact of the two Illumina methods on TPM values by direct comparison of datasets obtained for the same samples with both approaches. Apart from all histone‐encoding RNAs, we found only few transcripts of annotated coding genes with significant differences among paired samples. These genes were all excluded from further analyses (*CCR2, CD28, CD84, MALAT1, PLCG2, RMRP, TERC, WDR74* and *ZNF460* significantly lower or missing in poly(A)‐RNA based libraries; *CCN1, S100A14, SAA1, SAA2‐SAA4* and *SNCG* significantly lower or missing in total RNA‐based libraries). We also excluded all mitochondrial genes, mitochondrial ribosomal genes, ribosomal (40S/60S) genes and non‐protein‐coding genes (*MIR, LINC*). After exclusion of these genes the data for all samples were renormalised. Comparability of Illumina full‐length mRNA data in TPM and Lexogen Quantseq 3′ focused data in CPM was ascertained by quantile–quantile plot.

### 2.16 Adjustment of RNA‐Seq data for contaminating cells

To adjust RNA‐Seq data for contaminating cells we used our previously published approach^25^ consisting of two consecutive steps: (i) estimation of the extent of contamination and exclusion of highly contaminated samples, and (ii) based on this estimation, adjustment of the remaining RNA‐Seq dataset by a linear model. To avoid ‘false‐positive’ results for genes highly expressed in a particular cell type we modified the original approach by omitting the previously introduced bias towards an underestimation of contaminations.^25^ In addition, we assumed a minimal percentage of contaminating cells as detailed further below.

In the first step, we manually identified references samples, that is samples with the lowest levels of contaminating marker RNAs. For this purpose, we defined cell‐type‐specific marker genes by applying the following criteria: (i) high expression of the marker gene in the target cell type (median TPM > 100 for TU, TAM and TAT; TPM > 1000 for ADI and MESO; TPM > 25 for CAF); (ii) maximum 10‐fold difference between minimum and maximum expression (median TPM) in any sample of the target cell type; and (iii) a ratio > 10 between the minimum TPM value in the target sample set and the minimum TPM values of all other sample sets. Markers were ranked by the latter parameter and the top two marker genes from different gene families were selected (Table S3). These markers were *EPCAM* and *CLDN4* for TU, *FCER1G* and *FCGR2A* for TAM, *TRAT1* and *THEMIS* for TAT, *LPL* and *ADIPOQ* for ADI, *ITLN1* and *HAS1* for MESO and *RAB3B* and *CDH4* for CAF. Most of these are either well established cell‐type‐selective markers, such as EPCAM, LPL and ADIPOQ, or have previously been mentioned in the context of the cell‐type‐specific functions, e.g., CLDN4,^43^ TRAT1,^44^ THEMIS,^45^ ITLN1,^46^, ^47^ HAS1^48^, ^49^, ^50^ and RAB3B,^51^ while CDH4 has not been described as a mesenchymal marker to date.

Using these markers, we defined reference samples as indicated in Table S3 and identified the following contaminating cells types to be the most relevant depending on the cell type of interest:

- ascTAM and ascTAT in samples of ascTU,
- ascTU and ascTAT in samples of ascTAM,
- ascTU and ascTAM in samples of ascTAT,
- omTAM, ascTAT, MESO and CAF in samples of omTU (ascTAT were used in place of omTAT which were not available in sufficient quantities),
- omTU, ascTAT and MESO in samples of omTAM,
- omTU, omTAM, MESO (and to lesser extent TAT) in samples of ADI,
- omTU, omTAM, ADI in samples of MESO,
- omTU, omTAM, ADI and MESO in samples of CAF.

To be able to determine the fraction of RNA from contaminating cells more precisely, we identified large marker gene sets composed of genes with (i) >50‐fold median fold change between the cell type of interest and the contaminating cell type and (ii) a median expression of >10 TPM in the contaminating cell type. Fold changes were calculated with an offset of 0.001 from 0 to avoid infinite values and enable ranking. Candidate genes were ranked by fold change, and the top 25 were chosen to determine the extent of contamination. This automated procedure selected the marker gene sets listed in Table S4 for all relevant combinations (see preceding paragraph) of cell types of interest and contaminating cell types.

Contamination (%) was then assessed for each marker gene in the respective 25‐marker gene set (as defined above) as

$$\frac{\left(TPM\;in\;target\;sample\right)-\left(TPM\;in\;target\;reference\;sample\right)}{\left(TPM\;in\;reference\;sample\;of\;contaminating\;cell\;type\right)}\times 100$$

and the median of the resulting 25 values was considered the percentage of contaminating cells in the sample analysed.

The outcome of this assessment is shown in Table S5 for ascites cells and in Table S6 for cells isolated from omentum. Assumed minimum contaminations (see above) are indicated by ‘ < …’ in both Tables S5 and S6. TU, TAM and TAT samples with ≥4% contamination and ADI samples with ≥6% contamination with any cell type were excluded from all subsequent analyses. None of the MESO and CAF samples were excluded. This revised set of samples was then subject to adjustment of TPM values using an algorithm based on our previously described linear model^25^:

$$\frac{\left(TPM\;in\;target\;sample\right)\;-\;\left(frac\;\times TPM\;in\;contaminating\;cell\;type\right)}{\left(1\;-\;frac\right)}$$

where *frac* is the fraction of the contaminating cell type, and *TPM in contaminating cell type* is the median calculated for the sample set of the contaminating cell type, or, if patient‐matched samples were available, the expression in those. Adjustments were performed iteratively for each contaminating cell type. Instances of negative corrected TPM values were set to 0. The complete adjusted and renormalised dataset is shown in Table S7.

### 2.17 Functional annotations

Functional annotations were performed by PANTHER gene ontology (GO) enrichment analysis (www.geneontology.org) to reveal associations of gene sets with biological functions. In case of redundancies in the search results only the term with the highest enrichment and significance was included. For gene upregulated in omTU cells the following specific terms were significantly enriched: ‘response to lipid’ (GO:0033993; fold enrichment = 7.5; FDR = 2e‐7), ‘regulation of response to stress’ (GO:0080134; fold enrichment = 4.2; FDR = 8e‐5), ‘apoptotic process’ (GO:0006915; fold enrichment = 5.3; FDR = 2e‐4) and ‘regulation of cell differentiation’ (GO:0045595; 3.9‐fold; FDR = 2e‐4). For gene upregulated in ascTU cells ‘mitotic cell cycle process’ (GO:1903047; fold enrichment = 5.9; FDR = 4.6e‐36) and related terms were the most significant hits by far, followed by ‘cellular response to stress’ (GO:0033554; fold enrichment = 2.4; FDR = 8.3e‐13) on rank 29 and ‘regulation of programmed cell death’ (GO:0043067; fold enrichment = 2.2; FDR = 1.2e‐7) on rank 109. Upstream regulators analyses were performed using the Ingenuity Pathway Analysis (IPA) database.

Lists of previously published compilations of all growth factors/cytokines and their cognate receptors^52^ were updated using the information the GeneCards database (http://www.genecards.org) and PubMed (Table S8). Selective ligand–receptor interaction within the EGFR, FGFR, TGFβ, SEMA and WNT families were derived from published studies and reviews.^53^, ^54^, ^55^, ^56^, ^57^ Based on these updated data we redefined groups of growth factor/cytokine receptors and their interacting ligands. A list of 858 proteins associated with ECM reorganisation (Table S9) was assembled by using the Ensembl database and searching for genes with the terms ‘extracellular matrix’, ‘collagen’, ‘integrin’ and ‘adhesion’ in their name or descriptions, annotated as secreted or membrane proteins in the Human Protein Atlas as previously described^57^ and not overlapping with lists of growth factors, cytokines and their receptors in Table S8.

### 2.18 Statistical analysis of experimental data

Comparative data were statistically analysed by paired or unpaired Student's *t*‐test (two‐sided, unequal variance), as indicated in the figure legends. False discovery rate (FDR) was determined by applying the Benjamini–Hochberg method to nominal *p* values determined by *t*‐test. Results were expressed as follows: *p < .05; **p < .01; ***p < .001; ****p < .0001. Box plots were constructed using the Seaborn boxplot function with Python.

### 2.19 Survival‐associated gene expression analysis

Associations between gene expression and relapse‐free survival (RFS) of ovarian cancer patients were analysed using the KM Plotter meta‐analysis database^58^ retrieved from http://kmplot.com (2015 version; serous OC; JetSet best probe), which contains the following 13 datasets: GSE14764 (*n* = 80), GSE15622 (*n* = 36), GSE18520 (*n* = 63), GSE19829 (*n* = 28), GSE23554 (*n* = 28), GSE26193 (*n* = 107), GSE26712 (*n* = 195), GSE27651 (*n* = 49), GSE30161 (*n* = 58), GSE3149 (*n* = 116), GSE51373 (*n* = 28), GSE9891 (*n* = 285) and TCGA (*n* = 565). Associations with overall survival (OS) were derived from the PRECOG database (https://precog.stanford.edu).^59^

## 3 RESULTS

### 3.1 Validity of RNA‐Seq data

According to our experience small fractions of contaminating cells are present even in samples with the highest enrichment, which is of particular concern where contaminating cell types have a much higher RNA content per cell, as may be the case, for instance, for tumour cells compared to host cells. Thus, a low percentage of contaminating cells could easily result in a high content of cell‐type specific RNA, and thus in ‘false‐positive’ results for genes highly expressed in contaminating cells. Besides excluding highly contaminated samples we adjusted the RNA‐Seq data for contaminating cells by making use of our previously published method,^25^ which uses estimated fractions of contaminating cells in linear modeling. To avoid the ‘false positives’ alluded to above, we omitted the previously introduced bias towards an underestimation of contaminations. We also assumed a minimal percentage of contaminating cells, since 100% purity cannot be achieved and minimal contaminations are extremely difficult to determine (see Section 2). By applying this adapted procedure weakly expressed genes are likely missed in cases where expression is orders of magnitude higher in another cell type. However, in view of the goal of the present study, we view such ‘false negatives’ as less concerning than the introduction of ‘false positives’, as low expressors are likely to have a limited impact within the tumour microenvironment in the presence of the high expressors.

Figures 2A and S1 illustrate the results of this approach. In the original dataset (Figure S1A), marker gene expression analysis showed no clear separation between the sample sets, indicating frequent cross‐contamination, for example contamination of omTAM and ADI samples with TU, of ADI samples with TAM and MESO and of omTU samples with TAM, MESO and CAF. This problem was completely abolished (relative expression in Figure 2A; TPM values in Figure S2B) by exclusion of heavily contaminated samples (> 6% of any contaminating cell type) and adjustment for contaminating cells as described above. This adjusted dataset was used for all subsequent analyses.

We next addressed the question whether RNA expression of cytokine and growth factor genes is a suitable parameter to predict the secretion of the corresponding proteins. Towards this goal we determined the proteome of conditioned medium (CM) from CAF by mass‐spectrometry‐based proteomics and compared the number of cytokines and growth factors found in the proteomes with the corresponding RNA‐Seq data. We chose CAF for this analysis, because CAF represent the least contaminated cell type. We detected *n* = 72 growth factors in the secretome and *n* = 209 in the transcriptome (TPM > 0.3), with an overlap of *n* = 70 (Figure 2B). The detection of these proteins in the secretome strongly correlated with the strength of RNA expression (Pearson's *r* = .77; Figure 2C and Table S9), which increased from 33% for weakly expressed genes (0.3 TPM) to 100% for strongly expressed genes (≥500 TPM), reflecting the previously reported different sensitivity of both methods.^17^ Furthermore, we observed a good correlation (Spearman's *ρ* = .61) between TPM values obtained by RNA‐Seq and LFQ values derived from MS‐based proteomics for all proteins in the CAF secretome (Figure 2D). Importantly, the observed clear correlation between RNA expression and the detection of secreted proteins provides strong evidence that RNA‐Seq data are a valid source for predicting the synthesis of cytokines and growth factors, and thus for the purpose of the present study.

### 3.2 Assessing the impact of cell‐type‐selective differences in mRNA content

Since tumour cells possibly contain higher amounts of RNA per cell than other cell types, their contribution to the HGSC secretome may be underestimated using normalised RNA‐Seq data. To address this issue we took the following approach: First, we identified genes with similar normalised TPM values across all tumour and host cell samples analysed. Among the genes with the lowest variance were *NDUFS2* and *NFSL1C* with a variability of <2‐fold across all samples and cell types. We then prepared RNA from equal numbers of TU and different host cells, and analysed *NDUFS2* and *NFSL1C* mRNA levels in these samples by RT‐qPCR without normalisation, so that differences in PCR signals directly reflect differences in mRNA content per cell. As shown in Figure 2E and F, mean Cy0 values for tumour cells were 1.5 higher in omTU compared to omTAM for *NDUFS2* and 0.5–1.0 for *NFSL1C*, indicating a maximally 2‐fold (1.0 Cy0) higher signal for omTU. For CAF, an approximately 2‐fold higher signal was observed compared to omTU. Figure S3 shows that signals increase linearly to input, thus validating the data shown in Figure 2E and F. Taken together, these findings suggest that differences in mRNA content are ≤2‐fold and therefore unlikely to exert a substantial influence on the interpretation of the RNA‐Seq data for the purpose of the present study, which focuses on markedly higher cell‐type‐selective differences in expression (see below).

### 3.3 The secretome of tumour and tumour‐associated host cells in HGSC

**FIGURE 3:**
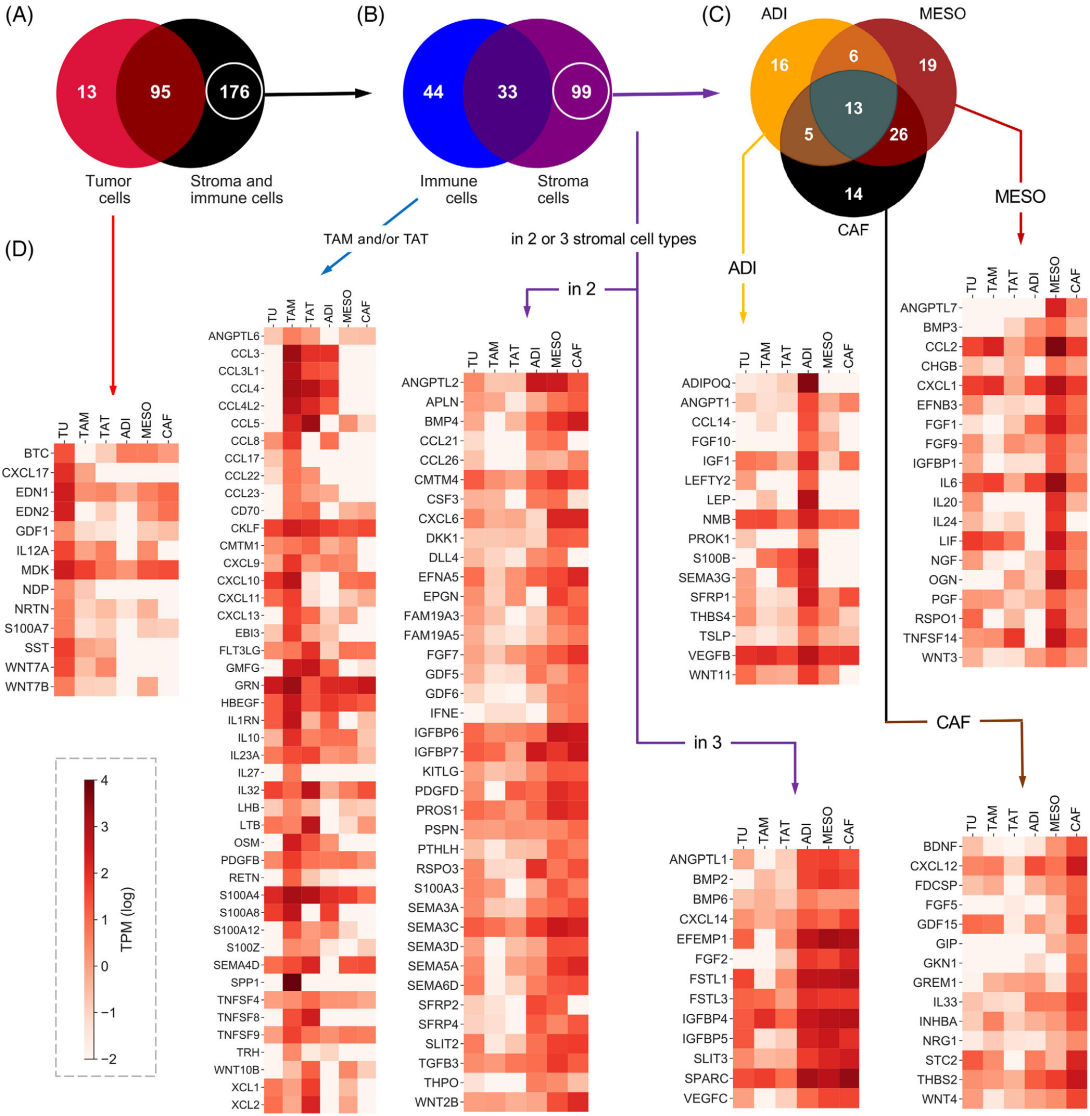
Expression of cytokine and growth factor genes in the omental TME. (A) Venn diagram showing the number of cytokine and growth factor genes expressed selectively in TU, selectively in stroma and immune cells or in all cell types (FC > 5). (B) Genes expressed selectively in stroma cells, selectively in immune cells or in both. (C) Genes expressed selectively in ADI, MESO, CAF or in combinations of these. (D) Expression patterns of compartment‐ or cell‐type‐selective genes in 6 different cell types of the omental TME. Expression levels are categorised (see bottom right) based on the values in Table S10

As the first step to decipher the signalling network of the TME of HGSC we determined which cytokine and growth factor genes are expressed by tumour cells and the most prominent host cell types in ascites and omentum. In total, we found *n* = 284 expressed cytokine and growth factor genes (TPM > 2). Unexpectedly, the majority of these genes (*n* = 176) were selectively (FC > 5) expressed in host cells (tumour‐associated immune cells and stromal cells of the omentum), whereas *n* = 13 genes were tumour‐cell‐selective and *n* = 95 genes were expressed in tumour and host cells with a less than 5‐fold difference (Figure 3A). Among host cells, omental stromal cells expressed a greater number of cytokine and growth factor genes than immune cells (*n* = 99 vs. *n* = 44; FC > 5; Figure 3B). Among stromal cells, ADI, MESO and CAF expressed similar numbers of genes in a cell‐type‐selective fashion (*n* = 16, *n* = 19 and *n* = 14, respectively), but also common sets of genes (e.g., *n* = 13 genes in all three cell types; *n* = 26 genes shared by MESO and CAF; Figure 3C). Figures 3D and S4 illustrate the expression patterns of compartment‐ or cell‐type‐selective genes in cell types of the omental TME based on the values in Table S10. These observations suggest that stromal cells of the omentum make a major contribution to the HGSC TME, in particular in view of the large number of ADI and MESO in the omentum or peritoneum.

### 3.4 Association of stroma‐selective cytokine and growth factor genes with patient survival

To assess the potential clinical relevance of the stroma‐selective cytokine and growth factor genes identified above we analysed their association with the relapse‐free survival (RFS) and the overall survival (OS) of ovarian cancer patients by interrogating two meta‐analysis‐based databases, the Kaplan‐Meier‐Plotter (KMP) database^58^ for RFS and the PRECOG database^59^ for OS. As shown in Table 1, 32 stroma‐selective genes showed a significant association with both RFS and OS [|*z*‐score| > 1.96]. Intriguingly, 31 of these genes were associated with a short RFS and OS. Only *BDNF* showed a significant association with a longer survival (HR < 1, *z*‐score ← 1.96). This result clearly points to a strong tumour‐promoting role for cytokines and growth factors produced by the stromal compartment of the omentum.

**TABLE 1:** Association of stroma‐selective cytokine and growth factor gene expression with RFS (KMP data base, *p*‐value and hazard ratio) and OS (PRECOG data; *z*‐score)

Gene	KMP (RFS) *p*‐value	KMP (RFS) HR	PRECOG (OS) *z*‐score	Cell type
*PDGFD*	.000002	1.55	5.53	MESO, CAF
*FSTL1*	.036424	1.20	5.09	ADI, MESO, CAF
*IGFBP6*	.014975	1.25	4.91	MESO, CAF
*THBS2*	.000001	1.57	4.59	CAF
*FGF1*	.000002	1.51	4.43	MESO
*WNT11*	.010436	1.25	4.42	ADI
*CXCL12*	.000010	1.51	4.39	ADI, CAF
*ANGPTL2*	.001481	1.32	4.36	ADI, MESO
*CXCL14*	.000903	1.36	4.29	ADI, MESO, CAF
*SPARC*	.000069	1.45	4.19	ADI, MESO, CAF
*SEMA3C*	.001502	1.31	3.91	ADI, MESO, CAF
*SFRP4*	.020009	1.25	3.90	ADI, MESO, CAF
*FSTL3*	.001730	1.32	3.75	ADI, MESO, CAF
*FST*	.000115	1.41	3.67	ADI, MESO, CAF
*ADIPOQ*	.000028	1.43	3.62	ADI
*THBS1*	.000169	1.41	3.57	ADI, MESO
*VEGFC*	.011139	1.26	3.56	ADI, MESO, CAF
*ANGPT1*	.001125	1.36	3.28	ADI
*SLIT3*	.000265	1.41	3.23	ADI, MESO, CAF
*IGF1*	.000002	1.51	3.18	ADI
*EFEMP1*	.009509	1.27	3.07	ADI, MESO, CAF
*GREM1*	.000165	1.38	3.05	ADI, CAF
*BMP2*	.007972	1.27	3.03	ADI, MESO, CAF
*SEMA5A*	.002967	1.29	2.96	MESO, CAF
*OGN*	.004469	1.30	2.80	MESO
*INHBA*	.000117	1.43	2.54	CAF
*IGFBP5*	.020092	1.22	2.42	ADI, MESO, CAF
*TGFB3*	.002885	1.32	2.36	ADI, MESO, CAF
*EFNB3*	.049981	1.20	2.32	MESO
*EFNA5*	.010729	1.28	2.21	ADI, MESO, CAF
*SFRP1*	.021237	1.26	2.03	ADI
*BDNF*	.004150	0.75	−3.02	CAF

A positive *z*‐score indicates a hazard ratio > 1, a negative *z*‐score a hazard ratio < 1; |*z*| = 1.96 corresponds to *p* = .05. The right‐most column indicated the main expressor cell type(s) among omental stromal cells based on the data in Figure 3. The Table shows only significant instances (*p* < .05 and |*z*| > 1.96)

### 3.5 Target cells of the HGSC secretome

**FIGURE 4:**
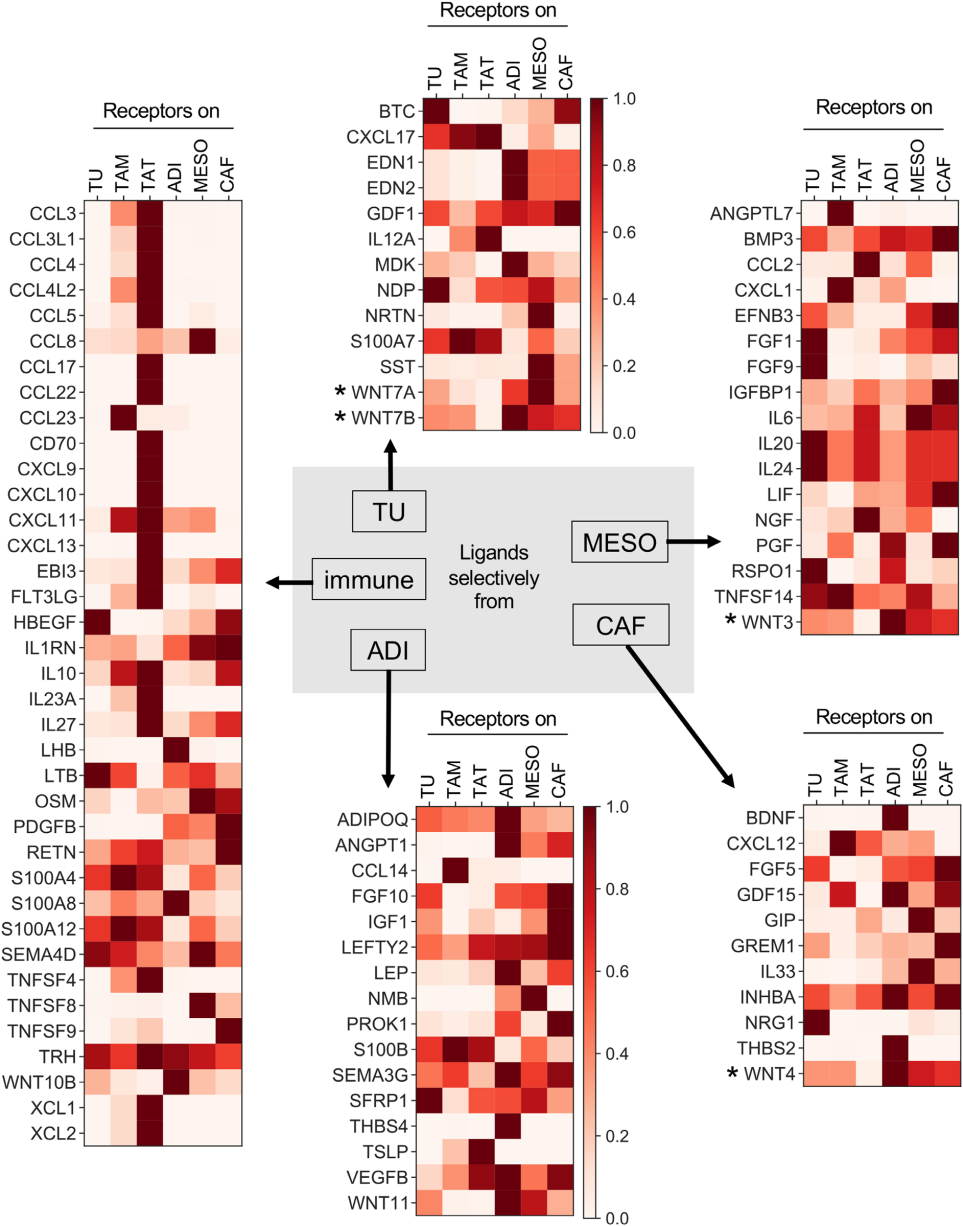
Targets of cell‐type‐selective ligands of the omental TME. The figure illustrates the expression of genes encoding receptors for the cell‐type‐selective ligands identified in Figure 2D. To take into account that numerous ligands bind to more than one receptor, we designed an algorithm that yields a normalised value for each ligand reflecting the relative expression of all receptor genes among different cell types. This was achieved by the following three consecutive steps: (i) TPM values were gene‐wise normalised for each cell type, (ii) normalised TPM values for all receptors of a given ligand were added up and (iii) the resulting values were normalised to cell type (scale bar top center; Table S11). *Wnt ligands and receptors constitute a highly complex relationship which obscure a simplified representation of the data. Therefore, data shown for WNTs are presumably skewed and should be treated with caution. Some of the genes in Figure 4 do not appear in this figure because their expression falls below the cut‐off of 2 TPM (e.g., *SPP1* from TAM)

We next sought to define the targets of cell‐type‐selective ligands of the omental TME. As many ligands bind to more than one receptor, the signalling network of the HGSC TME is extremely complex (Figure S5). To visualise the network for the ligands identified in Figure 3D in a comprehensible way we reduced its complexity by determining a normalised value for each ligand reflecting the relative expression of all receptor genes among different cell types (see legend to Figure 4 for details; complete dataset in Table S11). The heat maps (Figure 4) constructed from this dataset illustrate that many of these cell‐type‐selective ligands also display considerable target cell type selectivity

### 3.6 The metastasis‐associated signalling network of HGSC

**FIGURE 5:**
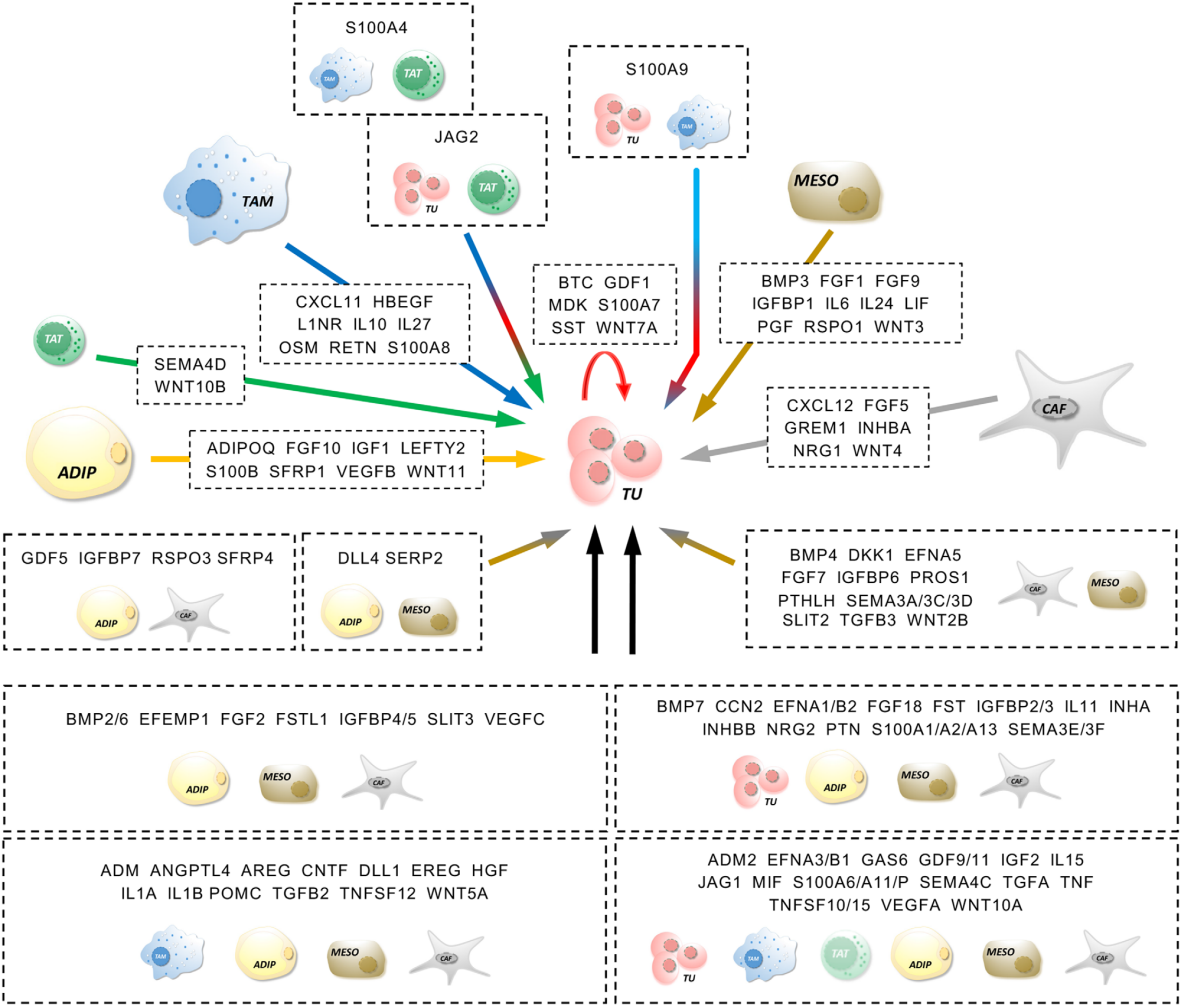
Schematic illustration of intercellular signalling pathways associated with metastasis and impinging on tumour cells. The figure incorporates all selectively expressed cytokines and growth factors previously linked to metastasis and ovarian cancer with receptors on omTU cells (TPM > 2; cell‐type‐selectivity threshold 5‐fold; as in Figures 2 and 3; Table S12)

Next, we directed our attention to intercellular signalling pathways targeting tumour cells and potentially linked to the metastatic spread of HGSC. To this end, we first retrieved all cytokine and growth factor genes previously mentioned in the literature in the context of both metastasis and ovarian cancer from the genecards.org database. Next, we identified a subset (*n* = 200) of these genes expressed in at least one cell type of the HGSC TME. For *n* = 122 of these genes cognate receptors were expressed by omTU cells (TPM > 2; Table S12). This dataset was used to construct the signalling map in Figure 5, ordered by ligands synthesised by single cell types or by groups of 2, 3, 4 or all 5 cell types. Notably, a substantial number of these gene is associated with a short survival, including *ADIPOQ, BMP2, CXCL12, EFEMP1, EFNA5, FGF1, FST, FSTL1, GREM1, IGF1, IGFBP5, IGFBP6, INHBA, SEMA3C, SFRP1, SFRP4, SLIT3, TGFB3* and *VEGFC* (Table 1). The data clearly underscore the prominent role of the tumour‐associated host cells, and in particular of omental stromal cells, in establishing a pro‐metastatic cytokine‐ and growth‐factor‐driven signalling network in HGSC.

### 3.7 WNT4‐mediated cross‐talk in the omental TME

**FIGURE 6:**
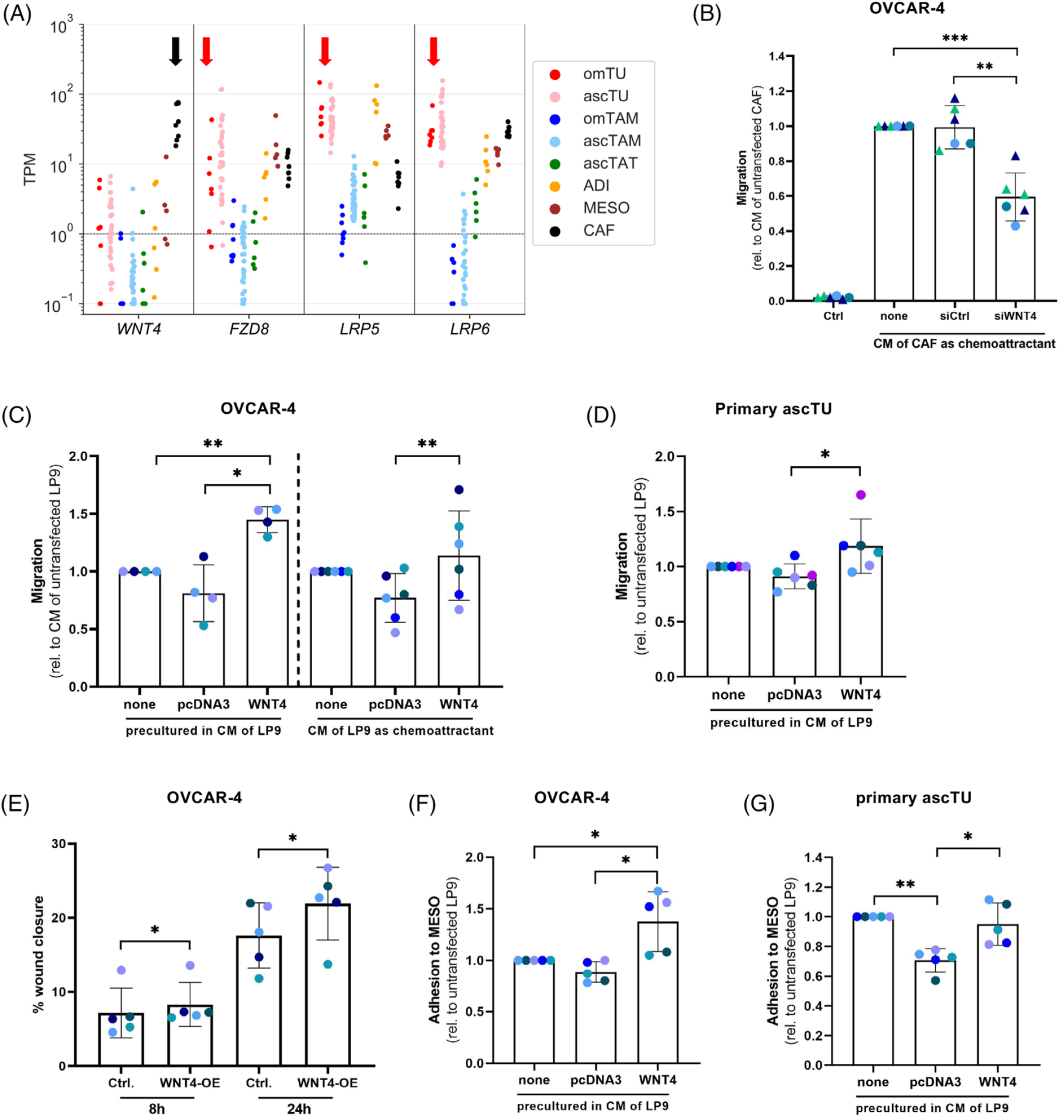
WNT4 signalling pathway and function. (A) Expression of the genes coding for WNT4, the major WNT4 receptor FZD8 and the FZD8 coreceptors LRP5 and LRP6 in different cell types from HGSC ascites and omentum. The same samples as in Figure 1A were analysed. The arrows indicate the selective expression of *WNT4* in CAF and the main WNT4 receptors in tumour cells. (B) Promotion of tumour cell (OVCAR4) migration by CM from CAF and inhibition by siRNA‐mediated interference with WNT4 expression by CAF. Ctrl: no conditioned medium. CM was harvested from cultured CAF isolated from four different patients used as chemoattractant for OVCAR4 cells. CAF CM of two patients (blue and green triangles) were tested in two independent experiments. (C) Migration of OVCAR4 cells in response to secreted WNT4 tested in two Transwell formats. CM from WNT4‐overexpressing, control‐transfected (pcDNA3) or untransfected LP9 cells were either used as chemoattractant (*n* = 6) or for pre‐incubation of OVCAR4 cells prior to migration towards 10% FCS as the chemoattractant (*n* = 4). Migration was calculated relative to CM from untransfected LP9 cells. (D) Migration of primary ascites‐derived HGSC cells (ascTU) pre‐incubated with CM from WNT4‐overexpressing, from control‐transfected (pcDNA3) and from untransfected LP9 cells, respectively (*n* = 6). (E) Wound healing capacity of OVCAR4 cells after incubation with CM from WNT4‐overexpressing and control‐transfected (pcDNA3) LP9 cells for 8 or 24 h (*n* = 5). Results are expressed as percentage of wound closure. Adhesion of OVCAR4 cells (F) and primary ascTU cells (G) to a confluent monolayer of peritoneal mesothelial cells (MESO). Tumour cells were pre‐incubated with CM from WNT4‐overexpressing, from control‐transfected (pcDNA3) and from untransfected LP9 cells, respectively, and labelled with CellTracker Green. Adhesion of tumour cells to the MESO layer was evaluated in comparison to CM from untransfected LP9 cells after 1 hr of coculture (OVCAR4 *n* = 5) or 2 h of coculture (ascTU *n* = 5). Columns in panels B‐G represent the mean. Standard deviations are shown as error bars. Asterisks indicate *p* values determined by two‐sided, paired *t*‐test. *p < .05, **p < .01, ***p < .001. Representative images are shown in Figures S8–S11

We chose WNT4 as an example to study the function of a highly cell‐type‐selective ligand in the cellular crosstalk within the HGSC TME. *WNT4* is expressed at >10‐fold higher levels in CAF compared to all other cell types (see black arrow in Figure 6A). The main receptor for WNT4 is FZD8, which form trimeric ligand–receptor complexes with the coreceptors LRP5 or LRP6 to initiate signal transduction.^60^ The expression pattern of these receptors (red arrows in Figure 6A) suggests that tumour cells are a prime target of WNT4, which could play a role in metastasis‐associated signal transduction pathways and biological processes.

This hypothesis was confirmed by the data in Figure 6B (representative microscopic pictures in Figure S8), which shows a reduced migration‐inducing potential of CM from CAF after transfection with a siRNA against *WNT4* compared to control siRNA (siRNA Figure S6). To obtain direct evidence for the migration‐promoting function of WNT4, we established a WNT4 overexpression model by transiently transfecting the WNT4^low^ human MESO line LP9 with a WNT4 expression vector or empty pCDNA3.1 as control. As shown in Figure S7, WNT4 expression and secretion were significantly increased 48 h after transfection in WNT4‐transfected cells compared to both pCDNA3.1 and non‐transfected LP9 cells. Using conditioned media (CM) from these cells, we investigated the impact of WNT4 on tumour cell motility and migration as well as tumour cell adhesion to the mesothelial layer. For these assays we used the HGSC cells line OVCAR‐4, which strongly expresses the WNT4 receptor FZD8 and coreceptors LRP5/6, but low levels of the WNT4 ligand. The data in Figure 6C (representative microscopic pictures in Figure S9) show that CM from WNT4‐overexpressing cells significantly enhanced the migration of OVCAR‐4 compared to CM from pCDNA3.1 control cells. This effect of WNT4‐CM was observed in two different experimental setups, that is when used as chemoattractant or for pre‐incubation of OVCAR‐4 cells to stimulate subsequent migration towards FCS. In line with these observations, CM of WNT4‐overexpressing cells increased the motility of OVCAR4 cells leading to faster gap closure in a wound healing assay (Figures 6E and S10).

As the attachment of tumour cells to a mesothelial cell layer is thought to represent an important step for transmesothelial migration and subsequent metastasis formation, we investigated whether WNT4 max play a role in this context. As demonstrated by the data in Figures 6F and S11, pre‐treatment of OVCAR4 cells with WNT4 enhanced their attachment to a mesothelial cell layer. Due to the limited biological significance of results obtained with established cell lines, we additionally tested the effects of WNT4 on primary HGSC tumour cells directly obtained from ascites, which confirmed our findings with OVCAR4 cells, as CM from WNT4‐overexpressing cells induced stronger tumour cell migration (Figures 6D and S9B) and adhesion to mesothelial cells (Figures 6G and S11B) compared to CM from control transfected cells . These data are consistent with a pro‐metastatic function of WNT4 in HGSC via a direct communication between CAF and tumour cells.

### 3.8 A major contribution by omental stroma cells to ECM‐associated signalling and reorganisation

**FIGURE 7:**
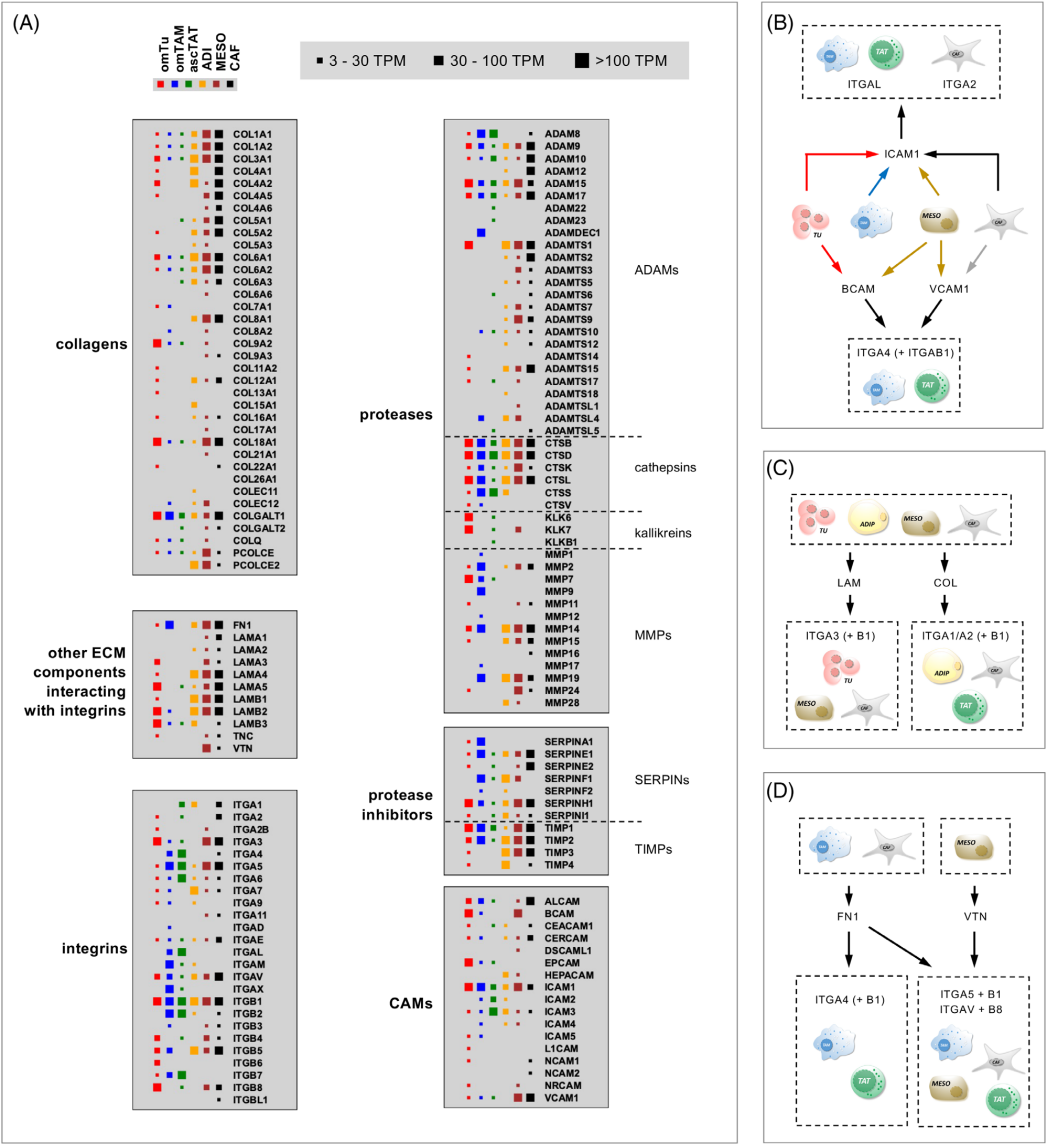
Expression of genes involved in ECM‐associated signalling and reorganisation. (A) RNA expression patterns in 6 different cells types categorised as indicated at the top. The figure is based on the values in Table S13. CAMs: cell adhesion molecules. (B–D) Schematic representation of major cell‐type‐dependent integrin‐mediated signalling pathways in the omental TME (examples) based on the data in panel A and published integrin‐ligand interactions.^99^, ^100^ The scheme depicts the cell types showing the highest expression of the respective gene or group of genes

The ECM plays a pivotal role in biological and molecular events linked to HGSC metastasis, such as cancer cell adhesion, migration and ECM‐mediated signalling. We therefore analysed the expression of ECM‐associated genes. For this purpose, we compiled a gene list from the Ensembl and Human protein Atlas databases (see Section 2 for details) and analysed the expression of these genes in our RNA‐Seq dataset (Table S13). The data for genes of particular relevance with respect to metastasis, that is ECM components, ECM‐remodeling proteins, cell adhesion molecules (CAMs) and integrins as ECM signal transducers, is summarised in Figure 7A (TPM > 2). It is obvious that omental stroma cells are the main producers of most collagens and, together with tumour cells, other ECM components (fibronectin, laminins, vitronectin). A different pattern was observed for ECM‐associated proteases and protease inhibitors, which are expressed by all cell types except TAT, but with clear gene‐selective patterns. For example, matrix metalloprotease genes, except for *MMP7*, are expressed at higher levels by TAM and stromal cells, with a high selectivity in case of *MMP9* and *MMP24* for TAM and CAF, respectively. Similarly, several protease inhibitors are expressed by most cell types (except TAT), such as SERPINH1 and TIMP1/2, while other are selective for TAM (*SERPINA1*), CAF (*SERPINE2)*, ADI (*TIMP4*) or all stromal cells (*TIMP3*).

As expected, integrins interacting with ECM components are expressed in a cell‐type‐selective fashion, such as *ITGA4, ITGAL* and *ITGB7* by immune cells, while tumour and stromal cells strongly expressed *ITGA3*, *ITGAV* and *ITGB1/4/5/6/8*. Major signalling pathways regulated by the interaction of integrins with ECM proteins or cell adhesion molecules are depicted in Figure 7B for BCAM and VCAM1 impinging mainly on TAM and TAT, for laminins interacting with tumour cells, ADI and MESO in (Figure 7C; left panel), for collagens with receptors on ADI, MESO and TAT (Figure 7C; right panel), or fibronectin interacting with TAM and TAT (Figure 7D; left panel), and for vitronectin preferentially signalling to immune cells, ADI and CAF (Figure 7D, right panel). Taken together, these observations point to an essential role of omental TAM and stromal cells in ECM‐mediated signalling pathways in the HGSC microenvironment.

### 3.9 Comparative analysis of tumour cells from ascites and omentum

**FIGURE 8:**
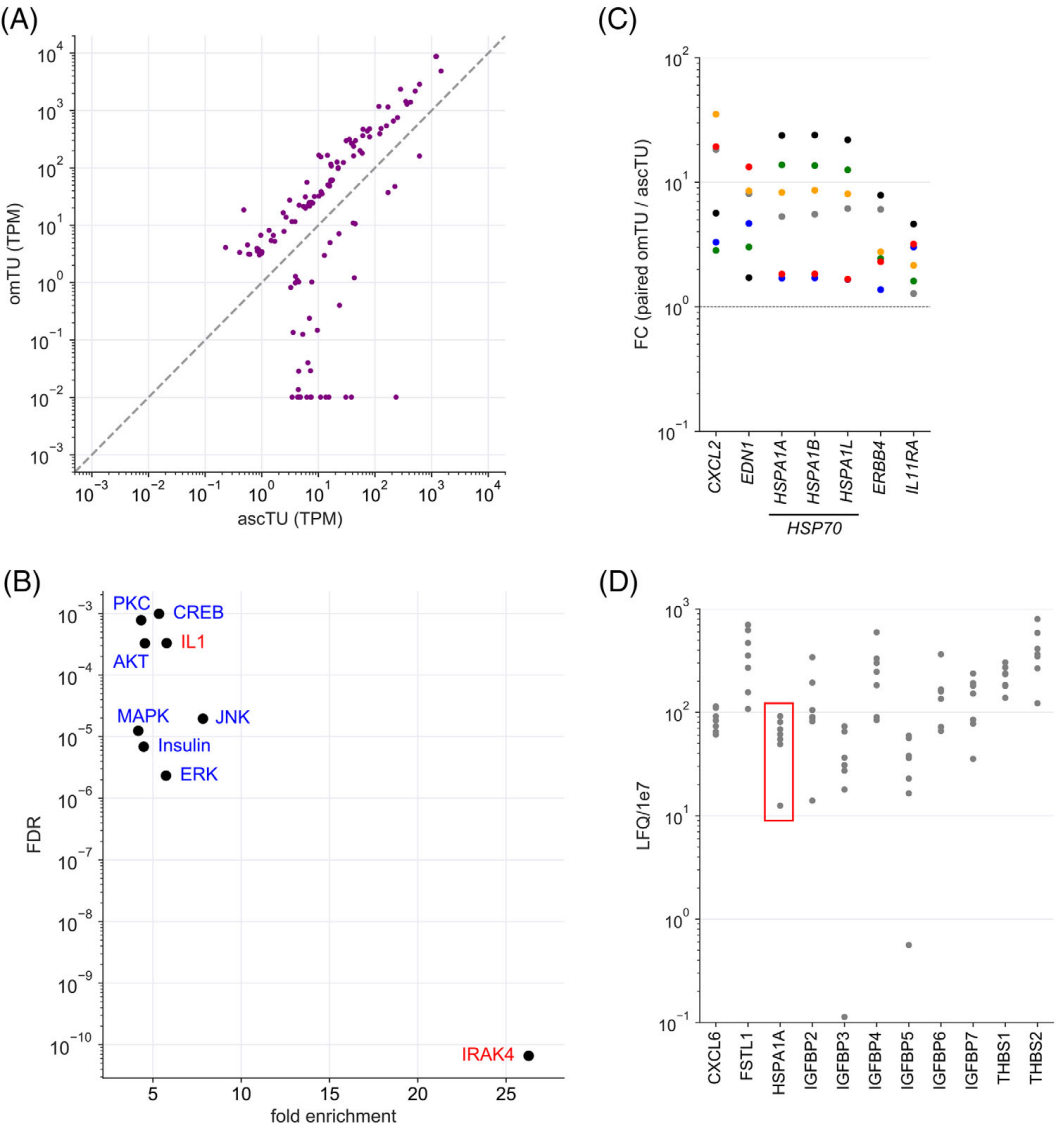
Signalling pathways of omTU compared to ascTU cells. (A) Expression of differentially expressed genes in omTU versus ascTU (FC > 3 and TPM > 3 in either cell type; nominal *p* < .05; Table S14). (B) IPA upstream regulator analysis of genes upregulated in omTU cells (*n* = 60; data points above diagonal in panel A). The plot shows the pathways with the highest significance (FDR < 0.001) and enrichment (>4‐fold) and a minimum number of enriched genes (*n* ≥ 6). (C) Regulation of genes coding for protein ligands in matched samples of omTU and ascTU cells (FC = TPM in omTU/TPM in ascTU). (D) MS‐based proteome analysis of conditioned medium obtained after a 16‐h culture of ascTU from 7 different patients. The plot shows HSPA1A (data points framed in red) and the 10 cytokines with the highest LFQ values

The differential contribution by tumour cell spheroids and by tumour cells in solid metastatic lesions to the signalling network of the TME has not been analysed to date. We therefore performed a systematic transcriptomic comparison of matched omTU and ascTU samples from 6 patients. As shown in Figure S12, the two sample sets showed a remarkably high correlation of *ρ* = .98, confirming that ascTU cells are an excellent source to obtain large numbers of tumour cells to study HGSC biology. Further analyses identified *n* = 121 significantly regulated genes (FC > 3; TPM > 3; nominal *p* value < .05 by paired *t*‐test), *n* = 83 genes with a higher expression in omTU versus ascTU samples and *n* = 38 genes with a lower expression in omTU (Figure 8A; Table S14).

Gene ontology (GO) term enrichment analysis of genes upregulated in omTU cells identified ‘regulation of cell differentiation’ as significantly enriched. In agreement with this finding, we found 52 out the 60 genes upregulated in omTU cells to be associated with ‘epithelial differentiation’ in the genecards.org database (Figure S13). Examples of epithelial genes consistently upregulated in all six samples include CLDN4, GJA1 and GJB1 (components of epithelial cell junctions), ERBB4 and ERRFI1 (EGF signalling), KRT16 (epithelial keratin) and the epithelial transcription factors KLF9 and KLF10. These observations point to enhanced epithelial‐like traits of omTU compared to ascTU cells. No significant enrichment of GO terms was observed with genes downregulated in omTU samples. Therefore, these genes were not further analysed.

Ingenuity pathway (IPA) upstream regulator analysis identified pro‐inflammatory (IRAK4, IL1) and MAPK‐associates pathways as significantly enriched in the set of genes upregulated in omTU cells (Figure 8B). Consistent with this result, we found 31 of the genes upregulated in omTU cells to be associated with the term ‘pro‐inflammatory’ in the genecards.org database. Remarkably all 31 genes were also present in the set of epithelial‐differentiation‐linked genes (marked by dots in Figure S13), pointing to a potential connection between pro‐inflammatory signalling and differentiation. Since the growth of metastases from spheroids is believed to involve transitions between epithelial and mesenchymal cancer cells phenotypes, these results could be of particular interest.

The genes upregulated in omTU cells comprised only two cytokines/growth factors (*CXCL2, EDN1*) and two cytokine/growth factor receptors (*ERBB4, IL11RA*), indicating a modest effect of different environment settings (omentum, ascites) on the contribution of TU to the signalling network of the TME (Figure 8C). Intriguingly, the upregulated ligand‐encoding genes also comprised the *HSP70* family members *HSPA1A, HSPA1B* and *HSPA1L* (Figure 8C), which upon their release from cells are able to act as extracellular signalling molecule.^61^ HSP70 proteins are of particular interest in view of the highly significant association of a short RFS with high HSPA1A levels in HGSC ascites.^2^ In agreement with the RNA‐Seq data, the secretome of tumour cells from 7 different patients contained high levels of HSPA1A after culturing in 50% ascites for 24 h, with MS signals ranking among the top 10 cytokines (Figure 8D; Table S16).

### 3.10 Comparative analysis of TAM from ascites and omentum

**FIGURE 9:**
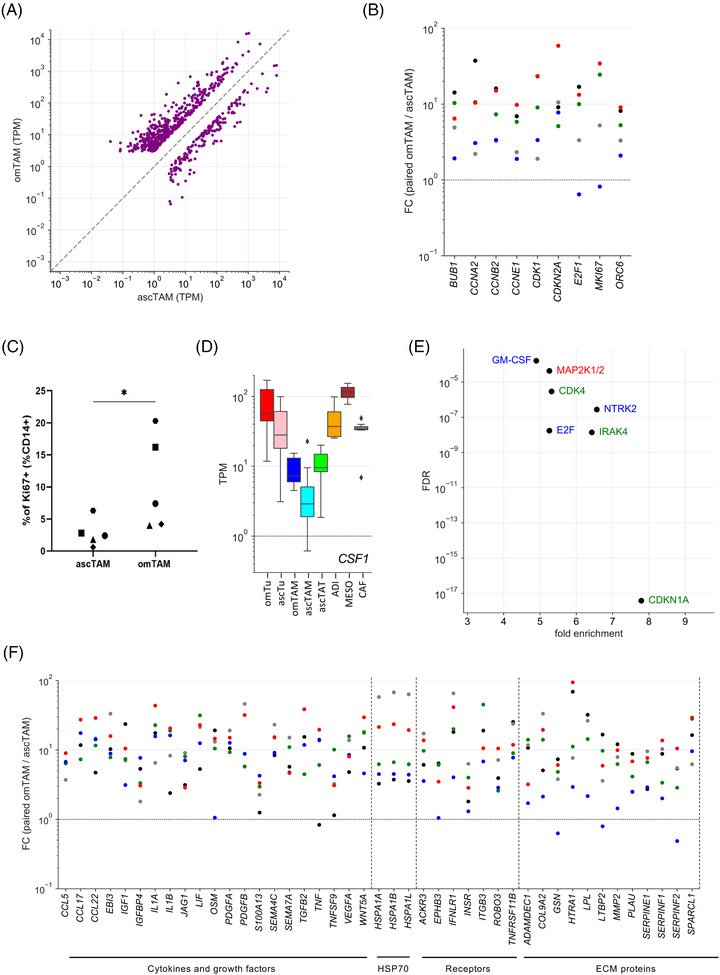
Signalling pathways of omTAM compared to ascTAM. (A) Expression of differentially expressed genes in omTAM versus ascTAM (FC > 3 and TPM > 3 in either cell type; nominal *p* < .05; Table S15). (B) Regulation of cell cycle genes in matched samples of omTU and ascTU cells (FC = TPM in omTU/TPM in ascTU). (C) Flow‐cytometric analysis of KI67 expression in matched omTAM and ascTAM samples from 5 HGSC patients (*p < .05; paired *t*‐test). (D) Expression of *CSF1* mRNA in different cell types from HGSC ascites and omentum. Boxplots show medians (horizontal line in boxes), upper and lower quartiles (boxes), range (whiskers) and outliers (diamonds). (E) IPA upstream regulator analysis of genes upregulated in omTAM (*n* = 456; data points above diagonal in panel A). The plot shows the pathways with the highest significance (FDR < 0.001) and enrichment (>4‐fold) and a minimum number of enriched genes (*n* ≥6). (F) Upregulation of genes coding for cytokines or growth factors, HSP70 family members, cytokine/growth factor receptors and ECM proteins in omTAM versus ascTAM (matched samples as in panel B)

We also compared the transcriptome of matched omTAM and ascTAM. Figure S14 shows a similarly high correlation (*ρ* = .95) as observed with tumour cells, but a larger number of differentially expressed genes (*n* = 674; TPM > 3; nominal *p* value < .05 by paired *t*‐test; Table S15). Of these, *n* = 516 genes were upregulated (FC > 3) in omTAM, and *n* = 158 were downregulated in omTAM (Figure 9A).

GO term enrichment analysis of genes upregulated in omTAM cells identified ‘mitotic cell cycle process’ and >50 other terms related to the cell cycle as highly enriched and significant. Figure 9A (examples) and Figure S15 (complete set) illustrate a consistent upregulation of all these genes in five out of six patient‐matched omTAM versus omTAM samples, including numerous CDKs, cyclins, E2F and its target genes and components of the mitotic spindle checkpoint. This finding clearly suggests that enhanced proliferation is a common feature of omTAM. This conclusion was confirmed by subsequent flow‐cytometric analysis of independent matched samples of omTAM and ascTAM, which revealed a clear increase in the fraction of KI67‐postive cells in five out of five omTAM samples analysed (Figure 9C). The main growth factor acting on macrophages is CSF1, which is strongly expressed in omental stroma cells, in particular in ADI and MESO (Figure 9D). In view of their abundance in the omentum it is very likely that these cell types are responsible for inducing the proliferation of omTAM via the secretion of CSF1.

In agreement with these observations, IPA upstream regulator analysis identified pathways directly associated with cell cycle progression (CDK4, CDKN1A, E2F) as well as their upstream signalling pathways (RAS, MAPK) as highly enriched in omTAM cells (Figure 9E). Significant enrichment was also observed for pro‐inflammatory pathways (IRAK4, GM‐CSF; Figure 9E) similar to omTU (Figure 8B), suggesting that the omental TME represents an inflammation‐promoting environment, which would be consistent with the presence of many pro‐inflammatory mediators (Figure 3).

In contrast to tumour cells (see above), omTAM expressed numerous genes encoding cytokines or growth factors (*n* = 18), HSP70 family members (*n* = 3), cytokine/growth factor receptors (*n* = 8) and ECM‐associated proteins (*n* = 13) at significantly higher levels as their counterparts in ascites (Figure 9F), providing further evidence for a major contribution of TAM to the omental TME. These include several genes coding for pro‐inflammatory cytokines (e.g., *CCL2, CCL17, CCL22, IL1A, LIF, OSM)*, and similar to omTU the HSP70 family genes *HSPA1A, HSPA1B* and *HSPA1L*.

Enrichment of GO terms was low or insignificant for genes downregulated in omTU samples. This gene set was therefore not subject to further analyses.

### 3.11 HSP70‐driven signalling

**FIGURE 10:**
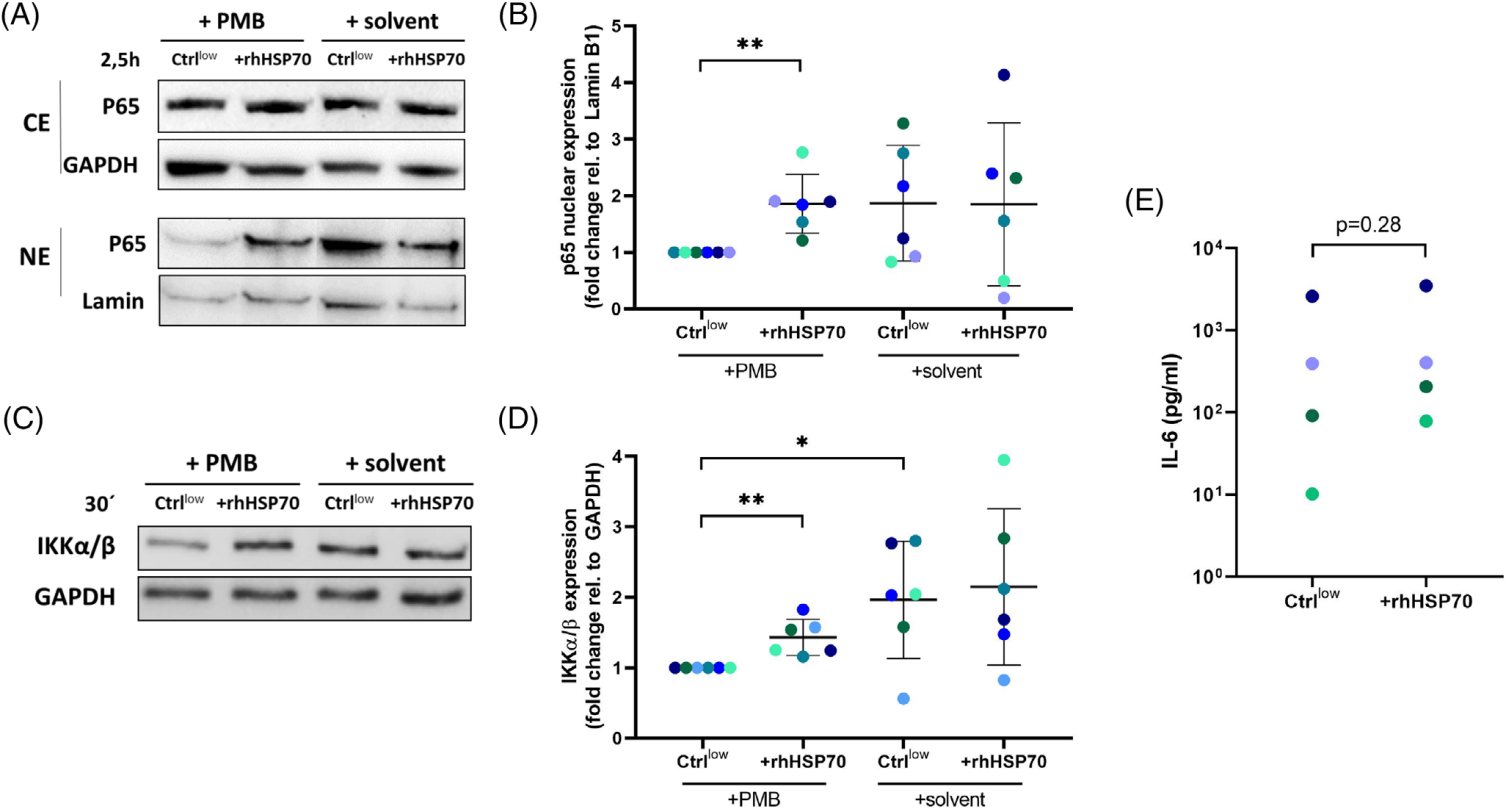
HSP70‐driven signalling in TAM. (A) Representative immunoblots showing induction of nuclear translocation of p65 by rhHSP70. NE: nuclear extracts; CE: cytosolic extracts. ascTAM were pre‐incubated for 2 h with 10 μg/ml polymyxin B (PMB) before 1 μg/ml low‐endotoxin rhHSP70 was added for 2.5 h. The control (Ctrl^low^) was spiked with the amount of LPS present in rhHSP70 as contaminant. (B) Quantification of nuclear translocation of p65 (relative to GAPDH, *n* = 6). (C) Representative immunoblot of whole cell lysates showing induction of IKKα/β (panel A) by rhHSP70. Conditions were as in panel A, except that rhHSP70 was added 30 min. (D) Quantification of IKKα/β expression (relative to GAPDH, *n* = 6). Each data point represents TAM from different patients. (E) Quantification of IL‐6 secretion by TAM after 24 h of rhHSP70 stimulation. IL‐6 in culture supernatants of TAM stimulated as described above were quantified by ELISA (*n* = 4 TAM). Horizontal bars indicate means. Asterisks indicate *p* values determined by two‐sided, paired *t*‐test. *p < .05, **p < .01

HSP70 can function as an extracellular ligand of toll‐like receptors (TLR2/4) and CD14 as coreceptor to trigger pro‐inflammatory signalling transduction^61^ and is associated with HGSC progression.^2^ We therefore investigated whether pro‐inflammatory NF‐κB signalling is activated by extracellular HSP70 in TAM which strongly express TLR2/4 and CD14 (Table S7). As shown in Figure 10A and B, stimulation of ascTAM (*n* = 6) with rhHSP70 resulted in significantly enhanced nuclear translocation of the NF‐κB p65 (RELA) subunit compared to a control (Ctrl^low^) containing LPS at a concentration corresponding to the contamination in low‐endotoxin rhHSP70. A potential contribution of endotoxin contaminations to NF‐κB activation was further minimised by treatment with the LPS inhibitor polymyxin B.^62^ Basal cytosolic levels of p65 remained unaffected indicating that only a minor fraction of cytosolic p65 translocates in the nucleus. In accordance with these results, increased levels of the IκB kinase IKKα/β was observed in all rhHSP70‐treated ascTAM samples tested (Figure 10C and D, *n* = 6), presumably as a consequence of decreased ubiquitin‐mediated degradation of IKKα/β.^63^ Finally, proinflammatory IL‐6 secretion by HSP70 was upregulated in rhHSP70‐stimulated ascTAM compared to the low‐endotoxin control Ctrl^low^ in three out of the four tested patients, albeit below statistical significance (Figure 10E). Taken together, these findings support the notion that extracellular HSP70 may contribute to metastasis by triggering the NF‐κB pathway and thereby a pro‐inflammatory response.

## 4 DISCUSSION

In the present study, we report the first intercellular signalling map for the TME of HGSC metastases, focusing on TU, TAM, ADI, MESO and CAF from the omentum as the primary site of transcoelomic dissemination of cancer cells. We also compared the intercellular signalling map of omental TU and TAM to their counterparts in ascites to work out commonalities and differences between these compartments. In view of the limitations of single‐cell sequencing for this purpose (see Section 1), we decided to perform these investigations by bulk RNA‐Seq analysis of purified cell populations obtained by protocols established and optimised as part of the present study.

### 4.1 Potential caveats of using RNA‐Seq data for the construction of an intercellular signalling network

A potential problem of the application of RNA‐Seq data to the construction of signalling networks is the extrapolation to protein levels, in particular with respect to secreted and membrane‐associated proteins, for which correlations with RNA levels are generally weak.^64^, ^65^ We addressed this issue using CAF in short‐term culture to obtain MS‐based secretome and RNA‐Seq data under the identical conditions (Figure 1C; Table S9). The results of this experiment showed a clear correlation (*r* = .77) between the percentage of cytokines and growth factors detected in the CM and the RNA‐Seq signal, reaching 100% for highly expressed genes. This correlation suggests that a fraction of secreted proteins (those expressed from more weakly expressed genes) is missing in the secretome due to a lower sensitivity of the proteomic analysis rather than intracellular regulatory mechanisms impinging on translation and/or secretion, consistent with previous data.^17^ Furthermore, many of the ‘missing’ proteins are membrane‐bound ligands, for example of the SEMA and TNFSF families, which is likely to result in low levels of soluble secreted protein (Table S9).

Another caveat potentially arises by using sample‐normalised RNA‐Seq data for the comparison of different cell types, if the latter contain highly divergent amounts of total RNA per cell. Thus, similar (normalised) TPM values could potentially obscure large differences in the absolute levels of a specific RNA (and thus of the encoded secreted protein) per cell. However, we could largely exclude this problem for the cell types analysed in the present study, since non‐normalised data from RT‐qPCR experiments showed a low variation across all samples and cell types for genes with a low variability (≤2‐fold) in the RNA‐Seq dataset (*NDUFS2* and *NFSL1C;* Figure 1D). We therefore conclude that differences in mRNA content are unlikely to impact the use of RNA‐Seq data for the focus of the present study.

Finally, cells isolated from omentum or ascites are invariably contaminated with other cell types. In case of highly expressed cell‐type‐specific genes contaminations could result in misleading data for weakly or non‐expressing cell types. To eliminate such ‘false positives’, we estimated the contamination of each sample by using predefined marker gene sets and eliminated all samples with >4% of any contaminating cell type (>6% for ADI samples). RNA‐Seq data for the remaining samples were adjusted for contaminations by adapting a previously described linear model,^25^ which solved the potential problem of cross‐contaminations (Figure 1B).

Comprehensive bioinformatic analyses of this adjusted dataset revealed an unexpectedly strong contribution by host cells, in particular by stromal cells, to signalling events linked to metastatic spread and survival. To further validate the significance of these findings, we compared our results to publicly accessible scRNA‐Seq data. Very recently, data for cells from seven untreated HGSC tumours were published, and for each cluster of tumour and host cells, specific transcriptomic markers were identified.^26^ We could verify in the scRNA‐Seq dataset the cell‐type specificity of the majority of TU‐selective (7/12), immune‐cell‐selective (32/43) and CAF‐selective (8/13) cytokine genes identified in the present study (Figure S16), supporting the conclusions drawn from our data.

### 4.2 Contribution by immune cells to the metastasis‐associated intercellular signalling pathways in the omental TME

Previous studies have shown that TAM from HGSC ascites are characterised by a high degree of phenotypic and ontogenetic heterogeneity,^52^ and comprise functionally divergent subgroups.^17^ Of these, CD163^high^ TAM are of particular relevance, as they express metastasis‐promoting genes, such as *CCL18, CCL23, KITLG* and *VEGFB* ^17^ and are associated with rapid tumour progression and early relapse in HGSC patients.^18^ By contrast, CD163^low^ TAM are linked to immune surveillance and a favourable clinical course, consistent with a higher expression of T‐cell‐attracting CXCR3 ligands CXCL9‐11.^17^, ^18^ As indicated by the present study, a similar diversity appears to exist among omTAM: while TAM produce most of the immune‐stimulatory or immune‐regulatory CCL‐ and CXCL‐type chemokines and cytokines (Figure 3D; left panel) acting on T cells (Figure 4; left panel), such as CXCR3 ligands, other TAM‐derived mediators possess pro‐metastatic potential and primarily address TU or stromal cells, such as HBEGF, OSM, RTN and S100A8 (Figures 3 and 4). In this context, TAM‐derived CXCL11 deserves particular attention, since it seems to be able to exert either pro‐ and anti‐tumourigenic effects dependent on its target cell. Thus, the migration of TU cells expressing CXCR3 is stimulated by CXCL11,^66^ which in view of the present data may also be applicable to the omental TME of HGSC (Figures 3 and 4). Intriguingly, our data also suggest that some pro‐metastatic mediators (such as SEMA4D and WNT10B) with cognate receptors on TU are selectively expressed by ascTAT (Figure 4), pointing to a potential and hitherto unrecognised role for re‐educated T cells in HGSC dissemination.

### 4.3 Contribution by omental ADI to metastasis‐associated pathways

Numerous studies suggest that ADI within the omental TME may play an essential role in HGSC progression^67^ by promoting the homing and invasion of cancer cells, angiogenesis and chemoresistance via the secretion of adipokines, including IL‐6, IL‐8, THF, leptin, adiponectin and resistin.^10^, ^68^, ^69^, ^70^ Our study extends these previous data by adding additional mediators to the list of adipokines expressed by omental ADI, some of them with a high degree of selectivity compared to other cell types, for example, CCL14, IGF1, PROK1, SEMA3G and THBS4 (Figure 3). Our data also show that receptors for numerous adipokines are expressed by TU and have previously been linked to metastasis, such as ADIPOQ, FGF10, IGF1, LEFTY2, S100B, SFRP1, VEGFB and WNT11 (Figures 4 and 5), compatible with a role in promoting HGSC progression by ADI‐borne mediators.

ADI have also been reported to induce CD36 on ovarian cancer cells allowing for the uptake of fatty acids and the formation of lipid droplets, thereby contributing to peritoneal metastasis.^11^ Our data, however, are difficult to reconcile with this model, since the expression of *CD36* is invariably extremely low in omTU (0–0.14 TPM; Table S7), while *CD36* expression is readily detectable in omTAM (4–36 TPM) and very high in ADI (>964 TPM). It therefore remains to be investigated whether a CD36‐mediated pathway is relevant for the stimulation of omental metastasis in HGSC patients.

### 4.4 Contribution by omental MESO to metastasis‐associated pathways

Another major cell type of the peritoneal TME and the omentum is the mesothelial cell. Gerber and colleagues identified hypoxic MESO at milky spots secreting vascular endothelial growth factor (VEGFA) suggesting that factors produced by MESO promote the growth of metastatic tumour cells by inducing neo‐angiogenesis.^8^ Angiogenesis may also be promoted by senescent MESO, which secrete elevated levels of IL‐6 and TGFβ to stimulate the expression of pro‐angiogenic CXCL1, CXCL8, HGF and VEGF by tumour cells.^71^ However, comprehensive and unbiased studies of the secretome of cancer‐associated MESO ave not been reported to date.

Our study clearly suggests that MESO play an important role in the intercellular signalling network of the omental TME. We have identified 19 mediators selectively expressed by MESO (Figure 3). Of these, 10 factors previously linked to metastasis directly address TU, including BMP3, FGF1/9, IGFBP1, IL6, IL24, LIF, PGF, RSPO1 and WNT3 (Figures 4 and 5). Furthermore, MESO and CAF co‐express several other potentially metastasis‐promoting factors with receptors on TU, that is BMP4, EFNA5, FGF7, SEMA3A/3C/3D, SLIT2, TGFB3 and WNT2B (Figures 4 and 5). MESO also share the expression of >60 other pro‐metastatic cytokine and growth factors with various other cell types of the omental TME (Figures 4 and 5). These observations clearly support the view that factors secreted by MESO partake in promoting the formation of omental lesions and presumably dissemination within the peritoneal cavity, especially considering the abundance of MESO as major constituents of all serous membranes.

### 4.5 Contribution by omental CAF to metastasis‐associated pathways

CAF have long been recognised as crucial components of most solid tumours, including ovarian cancer.^14^ CAF are derived from omental fibroblasts and mesenchymal stem cells, which is partly triggered by TGFβ and LPA in the tumour microenvironment.^9^, ^13^ CAF produce numerous factors acting on cancer cells to promote glycolytic metabolism, proliferation, invasion, angiogenesis and metastatic colonisation, including CCL5, CXCL10, IL‐6, TGFα, SDF and versican.^9^, ^14^, ^15^ Our own data support the concept of an instrumental role for CAF in HGSC metastasis. Omental CAF are the main producers of 14 cytokines and growth factors (Figure 3C and D), of which at least 6 of these have been associated with metastasis and possess receptors on omTU, including CXCL12, FGF5 and WNT4 (Figures 4 and 5). Furthermore, CAF share many of the mediators synthesised by other stromal cell types (*n* = 39; Figure 3C), and also address other host cell types (such as TAM, MESO and ADI), providing further evidence for the relevance these stromal cell types in the pro‐metastatic HGSC secretome.

To address the functional significance of the crosstalk of CAF and omTU we selected WNT4 as an example of a mediator expressed by CAF with high selectivity. Several WNT ligands have been reported to be upregulated and associated with histological grade, EMT, chemoresistance, and poor prognosis in ovarian cancer.^72^, ^73^, ^74^ Moreover, a strong inverse correlation between WNT activity and intra‐tumoural T cell infiltration resembling an immunologically ‘cold’ TME, has been demonstrated.^75^ WNT4 is a highly interesting ligand due to its proposed pro‐metastatic functions in laryngeal and colorectal carcinoma,^76^, ^77^ but knowledge regarding its role in the TME of other entities, including HGSC, is lacking. This problem is partly due to the diversity of FZD receptor isoforms and coreceptors and the complexity of different canonical and non‐canonical signal transduction pathways, which in turn trigger different functional outcomes.^78^ For WNT4, reduced expression in ovarian tumour cells compared to normal ovarian tissue has been observed in one study,^79^ which agrees with our own data showing weak expression of WNT4 in omTU (Figure 6A). Based on our observations, CAF are highly selective producers of the WNT4 ligand in the peritoneal TME. This is in line with previous publications reporting high expression of WNT4 and activation of WNT4 signalling pathways in fibroblasts in wound healing and fibrosis.^80^, ^81^ In contrast to WNT4, FZD receptor subtypes and LRP5/6 coreceptors are widely expressed among all cell types of the omental TME (Figure 4), indicating that CAF‐derived WNT4 may act on different cell types. Tumour cells present in the TME, for example, express FZD8 and LRP5/6 coreceptors (Figure 6A) known to interact with WNT4 ligand.^60^ Consistently, we were able to demonstrate that WNT4 significantly enhances HGSC motility, migration and adhesion to a mesothelial layer (Figure 6), which underlines a potential role of WNT4 secreted by CAF in peritoneal metastasis.

### 4.6 Invasion of tumour cells into the mesothelium

The mesothelium consists of a single layer of MESO covering a basement membrane composed of extracellular matrix (ECM) proteins. According to the prevailing opinion, the mesothelium constitutes a barrier against the adhesion of, and invasion by, cancer cells from the peritoneal fluid.^82^ The attachment of cancer cells is thought to be dependent on pre‐existing lesions of the mesothelium to allow for an interaction of integrins on tumour cells with the underlying ECM proteins.^17^, ^83^, ^84^, ^85^, ^86^ Different models have been proposed to explain the occurrence of such lesions. Besides MESO senescence^87^ and myosin‐dependent mechanical forces exerted by tumour cells,^88^ cytokine‐mediated MESO activation^89^, ^90^ has been suggested as mechanisms facilitation penetration through the mesothelial layer. The latter is compatible with our data, which indicate that a number of mediators with receptors on MESO and the potential to activate MESO are produced by TU or host cells, including IL‐1A, IL‐1B, IL‐15, IL‐23A and TGFβ (Figure S4). Active MESO killing by FAS ligand^91^ is another mechanism proposed to enable the penetration of cancer cells through the mesothelial layer. According to our data, TAT express high levels of *FASLG* and MESO express the corresponding receptor gene (Figure S5), supporting the aforementioned hypothesis. However, other mechanisms involving death‐ligand‐mediated killing mechanisms are possible, as suggested by the expression of several other death receptors of the *TNFSFR* family by MESO, for example *TNFRSF8*, *CD40 (TNFRSF5)* and *CD27 (TNFRSF)*, which encode receptors for CD30 (TNFSF8), CD40LG und CD70 (Figure S5). Future work will address the hypotheses arising from these data.

Invasion of cancer cells is critically dependent on their interaction with collagen fibers beneath the MESO layer, consistent with the documented association of ECM modifiers with HGSC survival.^52^ As shown by our data, all cell types contribute to ECM reorganisation, but in a cell‐type‐selective manner and with a predominant role for TAM and stroma cells (Figure 7). This is evident from the fact that omTAM are also a major source of gene products involved of ECM reorganisation, including laminin, proteases of the ADAM, cathepsin and MMP subgroups as well as SERPIN and TIMP protease inhibitors. Stromal cells also strongly contribute to proteolysis and its regulation, but are the main producers of collagen and other ECM components. Taken together with previous findings that ECM remodeling is associated with ovarian cancer survival,^17^, ^52^ these observations provide further strong evidence for the relevance of TAM, ADI, MESO and CAF in the TME of HGSC.

### 4.7 Comparison of TU and TAM from ascites and omentum

In the present study, we also present the first comparative analysis of cell populations isolated from HGSC metastases and ascites. An important conclusion derived from this analysis is the high similarity of matched omTU/ascTU and omTAM/ascTAM samples with correlation coefficients of 0.98 and 0.95, respectively (Figures S12 and S14). Many previous studies made use of ascites cells for molecular and functional analyses, because these cells are available in large numbers and can be isolated as relatively pure fractions. Our data show that HGSC ascites cells faithfully reflect the respective cellular compartments of the TME and therefore validate their use for studying HGSC biology.

There are, however, also clear selective differences, as exemplified by the induction of cell cycle genes in omTAM (Figure 9B), which we were able to confirm by flow cytometry (Figure 9C). The induction of TAM proliferation is possibly due to their spatial proximity to omental stromal cells, which strongly express the *CSF1* gene (Figure 9D) encoding the essential macrophage‐specific growth factor M‐CSF.^92^ This observation also suggests that ascTAM arise from replicating resident macrophages (and blood monocytes) rather than from their proliferation in ascites.

Another marked difference between both omental and ascites cells is the upregulation of inflammatory signalling, which was seen with both TU and TAM (Figures 8B and 9E). Among others, HSPA1A is one of the pro‐inflammatory genes upregulated in both omental TU and TAM compared to their counterparts in ascites. Consistent with this observation, we found high levels of HSPA1A in conditioned medium of ascTU (Figure 8D). HSP70 can also be found at high concentrations in the ascitic fluid where its presence is linked to a poor RFS of HGSC patients.^2^ Besides acting as an intracellular molecular chaperone, HSP70 can be released from cells in case of elevated cellular stress.^61^ Extracellular HSP70 (eHSP70) provides danger signals to the immune system as a damage‐associated molecular pattern (DAMP) resulting in pro‐inflammatory activation of macrophages, monocytes or dendritic cells by inducing secretion of cytokines such as IL‐1, IL‐6 and TNF‐α.^61^, ^93^ By contrast, other studies point to an inverse immunoregulatory role of eHSP70 that dampens inflammation.^94^, ^95^ eHSP70 can exert cytokine regulatory activity by engaging TLR2 and 4 receptors, which in turn activate NF‐κB and MAPK signalling pathways,^61^ but the precise mechanism how eHSP70 acts on TU and TAM in the TME is unclear. Moreover, the activation of HSP70 through TLR2/4 is controversial, since contaminating LPS in the recombinant HSP70 used in one studies may account for its pro‐inflammatory activity.^96^ Another study reported that the stimulatory effect requires both, the presence of endotoxin and structural integrity of HSP70.^97^ By using highly purified, low‐endotoxin rhHSP70 in conjunction with polymyxin B treatment to suppress LPS‐mediated TLR4 activation, we were able to detect activation of the NF‐κB pathway by increased nuclear p65 translocation and induction of IKKα/β in TAM of different patients (Figure 10A and B), accompanied by an upregulation of IL‐6 secretion (Figure 10C). Multiple pro‐tumourigenic functions have been assigned to IL‐6 in ovarian cancer including invasion, migration, EMT, proliferation, overexpression of metalloproteases and chemoresistance.^98^ Thus, induction of pro‐inflammatory signalling (including IL‐6 secretion) via upregulation of HSP70 in omTU and/or omTAM might be involved in promoting the peritoneal spread of cancer cells in HGSC patients.

### 4.8 Conclusions

Factors secreted by immune and stroma cells contribute to an unexpected large extent to the intercellular signalling network of omental metastases and establish an environment that supports HGSC progression. Intriguingly, the expression of numerous genes coding for cytokines, growth factors and ECM remodeling proteins within this network are associated with tumour progression, metastasis and survival, pointing to their relevance as potential biomarkers and/or targets for therapeutic intervention. As proof of principle, we demonstrate a tumour‐promoting function of the highly cell‐selective mediator WNT4 produced by CAF and acting on tumour cells to induce their migration. In spite of a generally high similarity between ascites‐derived cell types and their counterparts in solid tissue, a selective shift towards a pro‐inflammatory gene expression pattern is activated in omTAM and omTU, probably triggered by interaction with adjacent stroma cells in the metastatic niche. Our data suggests that the omental TME represents an inflammation‐promoting environment which might be linked to transitions between epithelial and mesenchymal cancer cell phenotypes as a prerequisite of metastatic growth.

## CONFLICT OF INTEREST

The authors declare that there is no conflict of interest.

## Supporting information



Figures S1–16

Tables S1–15

Supporting Information
