# Supplementary material for: The multicellular signalling network of ovarian cancer metastases
Source: Clin Transl Med. 2021 Nov 8;11(11):e633. doi: 10.1002/ctm2.633 (PMC8574964; doi:10.1002/ctm2.633)
Supplement: Supplementary file 1 — Figures S1–16 [file CTM2-11-e633-s002.pdf]

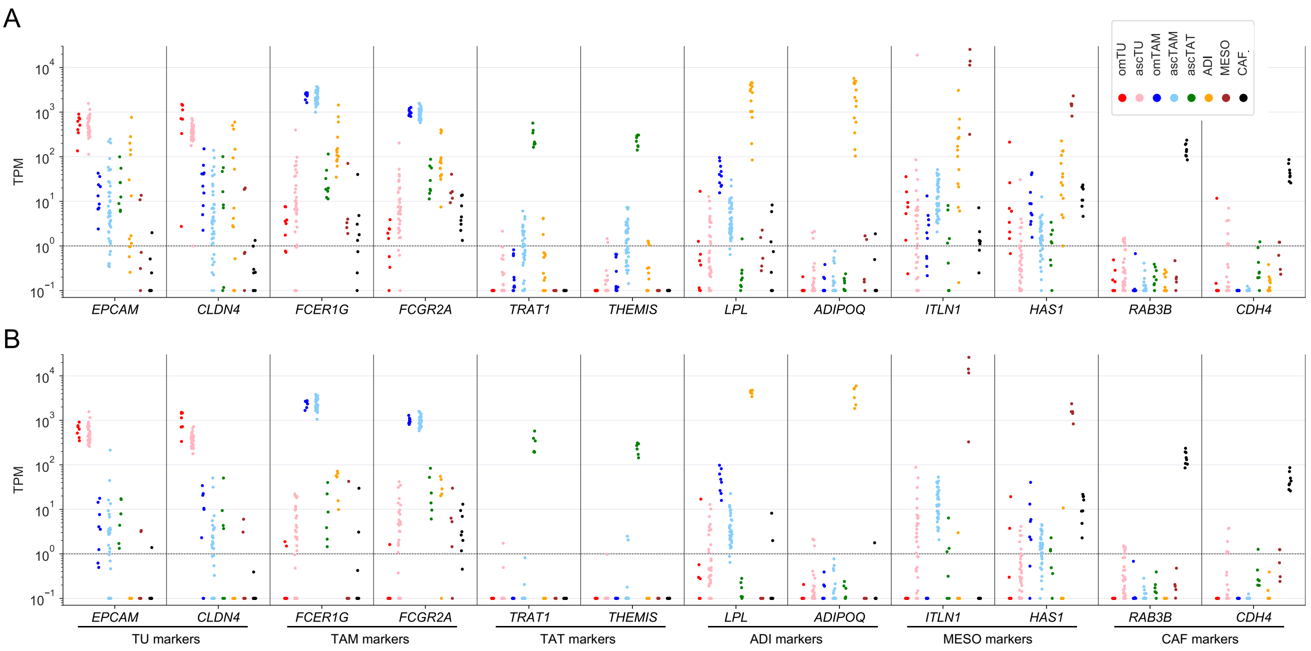

FIGURE S1

(A) Expression (TPM) of cell type-specific marker genes in all samples isolated from HGSC ascites and omentum determined by RNA-Seq. (B) Expression (TPM) of the same markers after exclusion of samples with >6% of any contaminating cell type and bioinformatic adjustment for contaminating cells. Samples with >6% of any contaminating cell type were excluded prior to adjustment. Each data point represents a different patient.

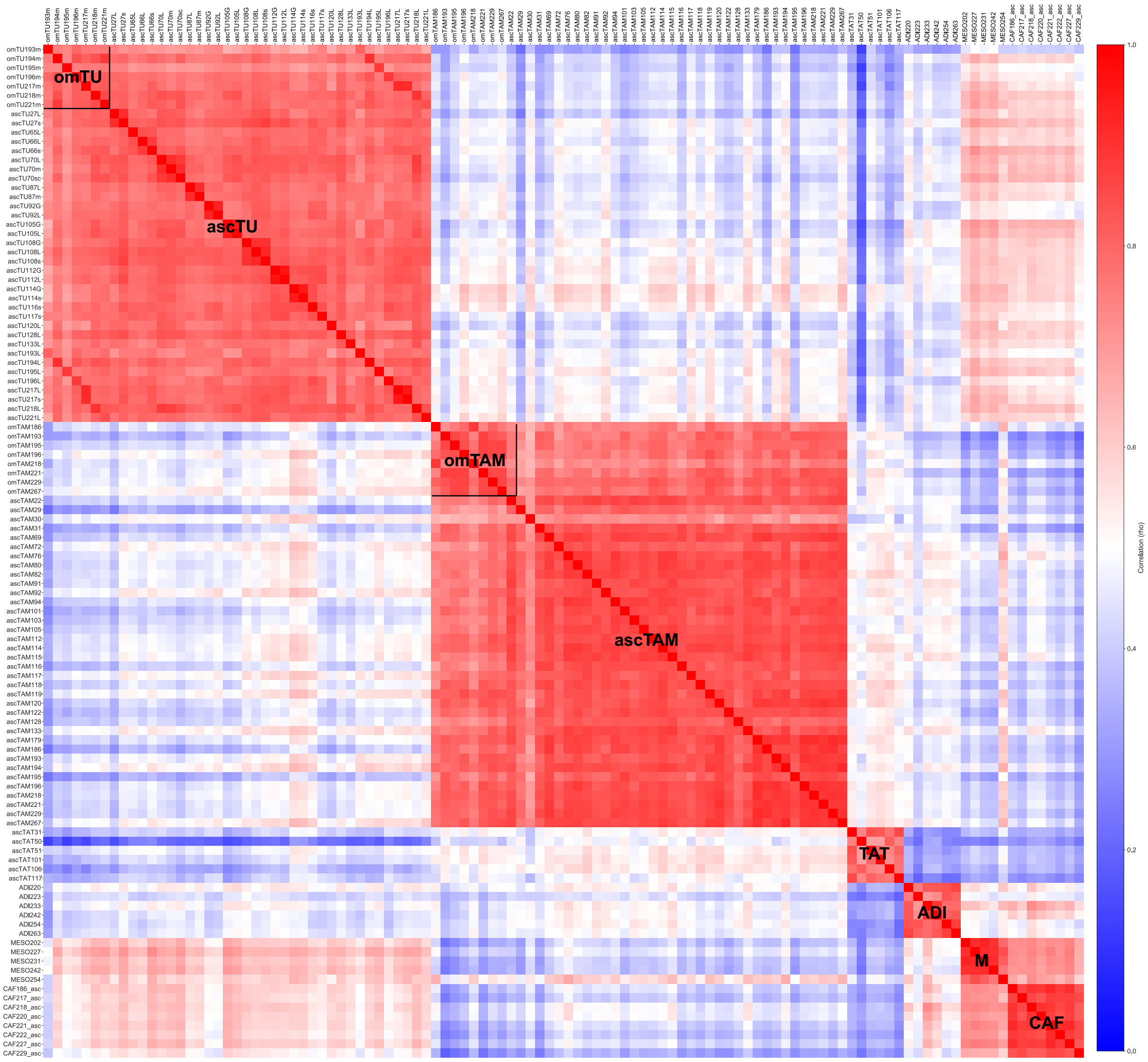

FIGURE S2

Heatmap showing the correlation (Spearman ρ) between all samples analyzed in the present study. The numbers in sample names represent the patient IDs, as listed in Table S1. Samples are ordered by cell type.

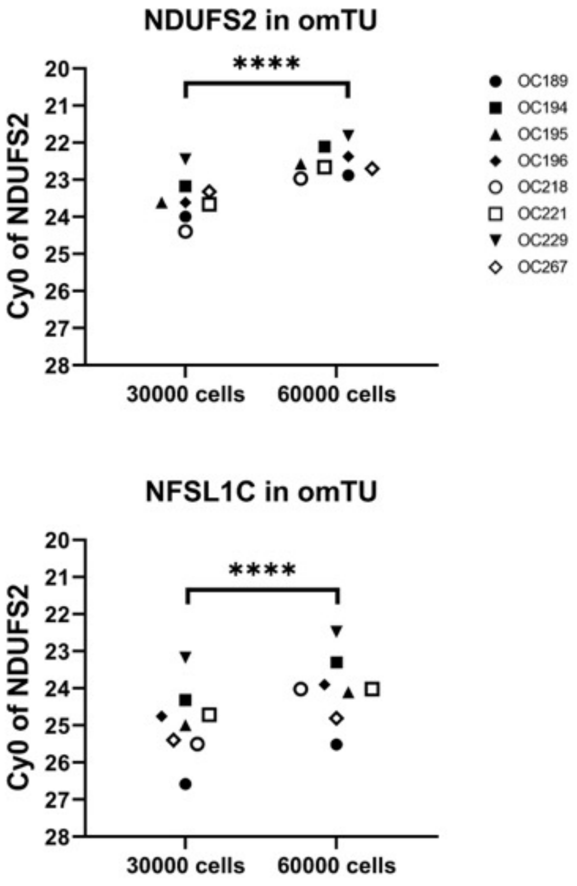

FIGURE S3
qRT-PCR data confirming a linear relationship between input and signal for the experiment in Figure 1D and E. RNA from different numbers of omTU cells was analyzed using primers for *NDUFS2* and *NFSL1C* mRNA without normalization. Each symbol indicated a different patient.

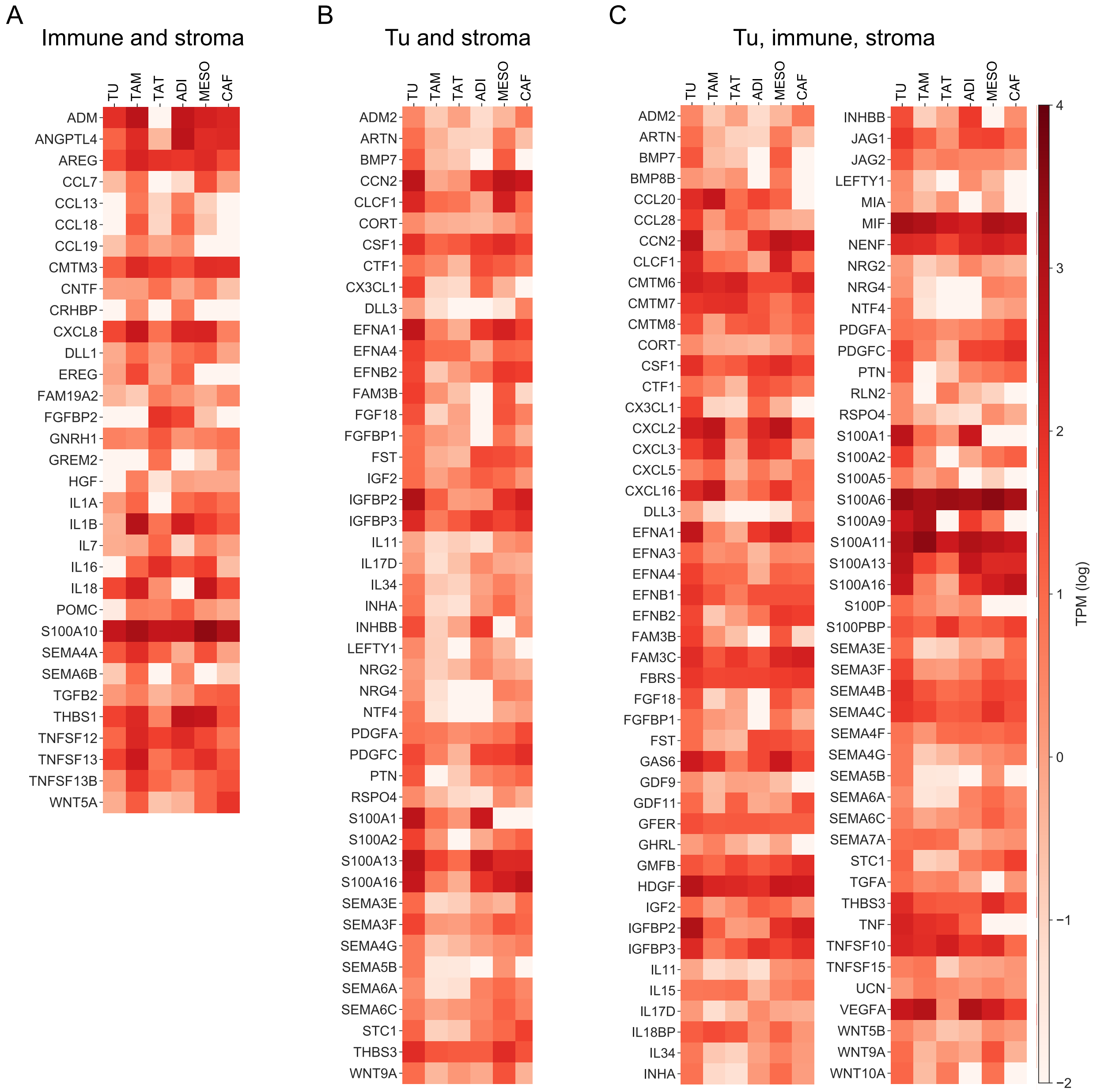

FIGURE S4

Expression patterns of genes encoding cytokines and growth factors in different compartments of the omental TME. Expression levels are categorized (see bottom left) based on the values in Table S10 (as in Figure 2C).

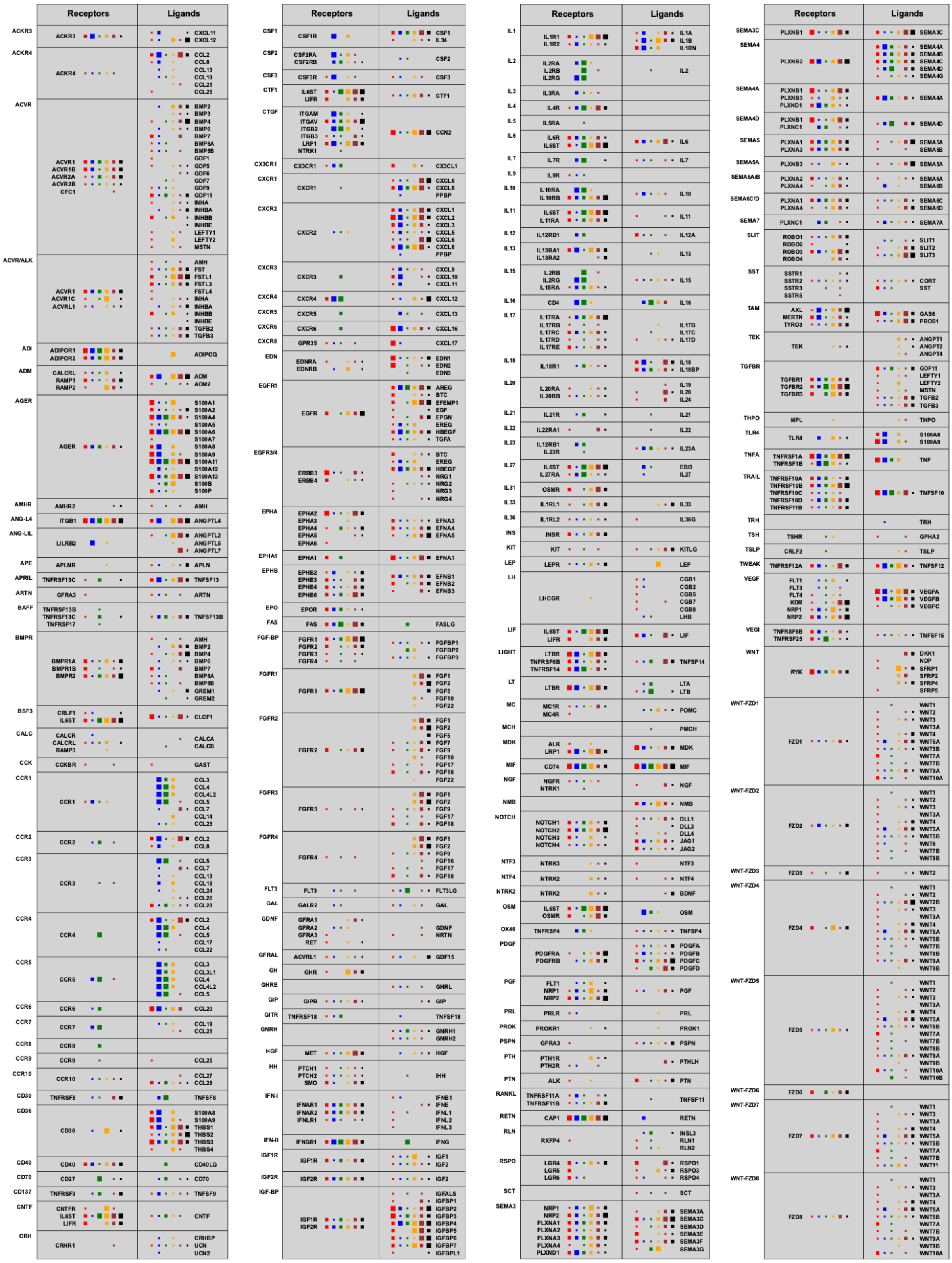

FIGURE S5

Expression patterns of genes encoding cytokines and growth factors in different compartments of the omental TME. Expression levels (medians; Table S7) were categorized as shown on the right.

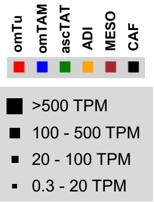

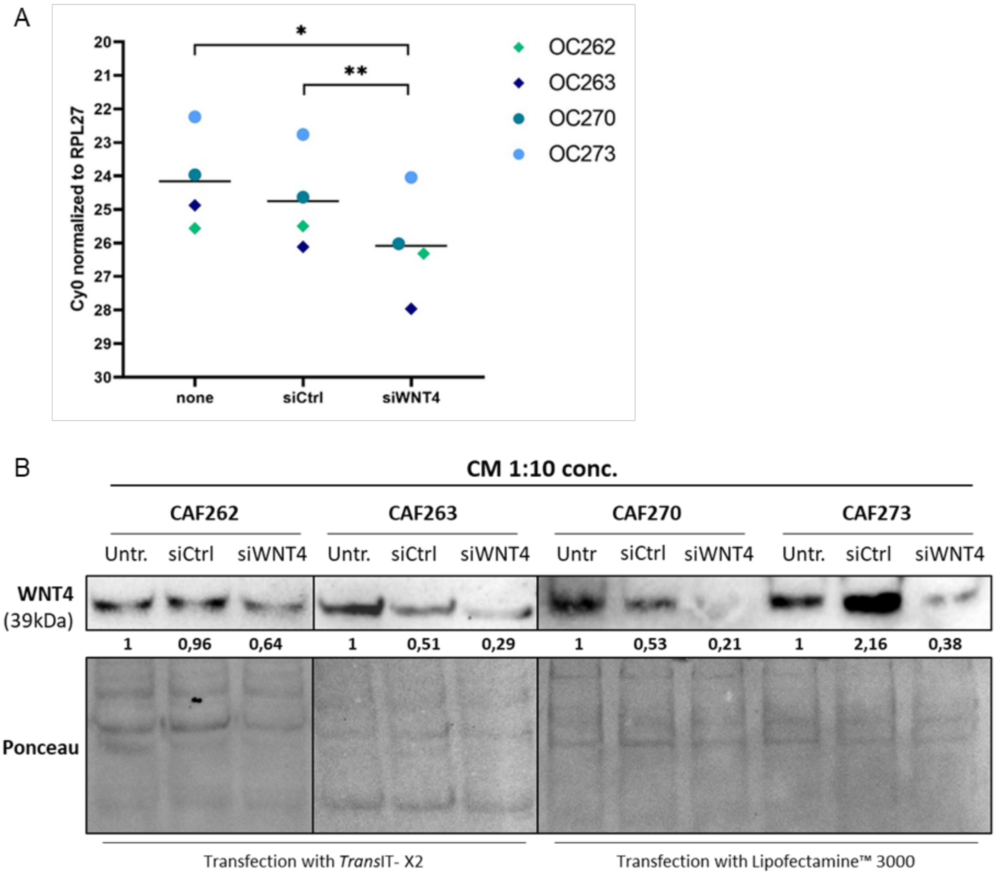

FIGURE S6

Confirmation of siRNA-mediate knockdown of WNT4 expression in CAF from four different patients using two different transfection reagents (CAF262, CAF263: TransIT-X2; CAF270, CAF273: Lipofectamine 3000). **(A)** qRT-PCR analysis of untransfected CAF and CAF transfected with control siRNA or WNT4-directed siRNA. Horizontal bars indicate means. Asterisks indicate p values determined by two-sided, paired t test. $^{*}p < 0.05$, $^{**}p < 0.01$. **(B)** Immunoblotting analysis of conditioned medium (CM, 1:10 concentrated) from the same samples as in panel A. Densitometry quantification of WNT4 signals are expressed relative to CM of untransfected CAF. Ponceau red staining demonstrates equal protein loading.

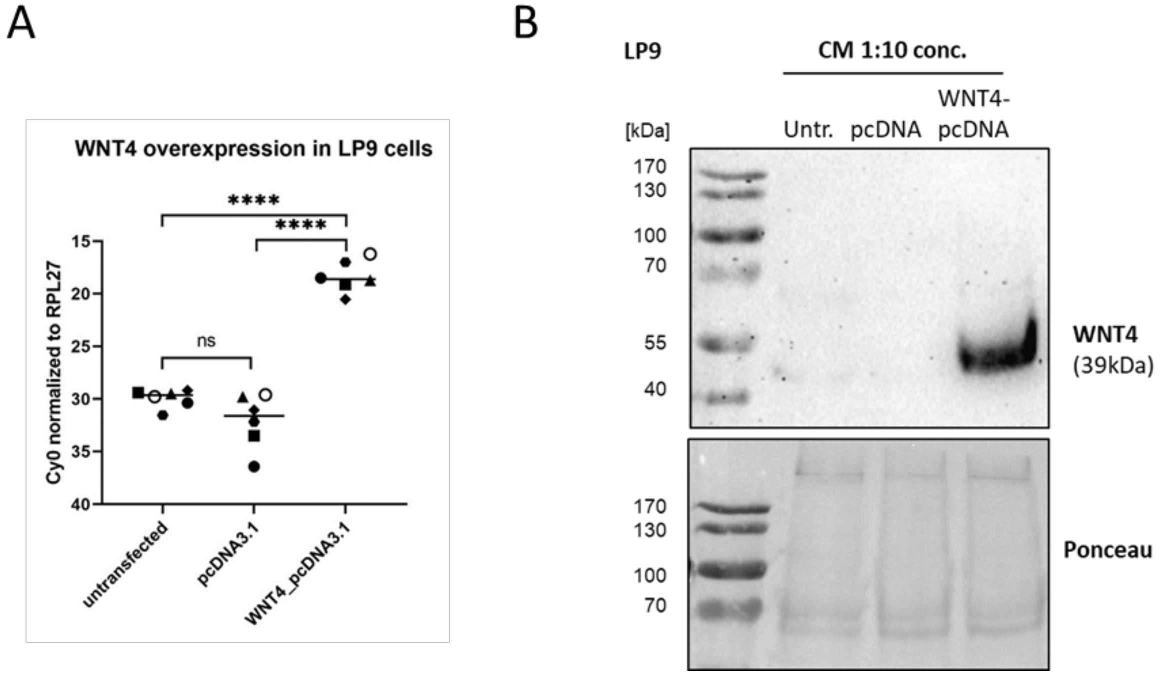

FIGURE S7

Confirmation of WNT4 overexpression in LP9 cells transiently transfected with WNT4-pcDNA. **(A)** Validation of *WNT4* expression in LP9 cells by qRT-PCR. Expression was analyzed 48h after transfection with WNT4-pcDNA or pcDNA3.1 control. Untransfected cells were included as additional controls. Data were normalized to *RPL27*. Each data point represents an individual experiment and horizontal bars indicate mean. p values were determined by two-sided, paired t test (****p < 0.0001). **(B)** Representative immunoblot showing WNT4 secretion in conditioned media (CM; 10-fold concentrated) of LP9 cells transfected with WNT4-pcDNA compared to pcDNA3.1 control and untransfected cells. Loading of equal amounts of protein was verified by Ponceau red staining.

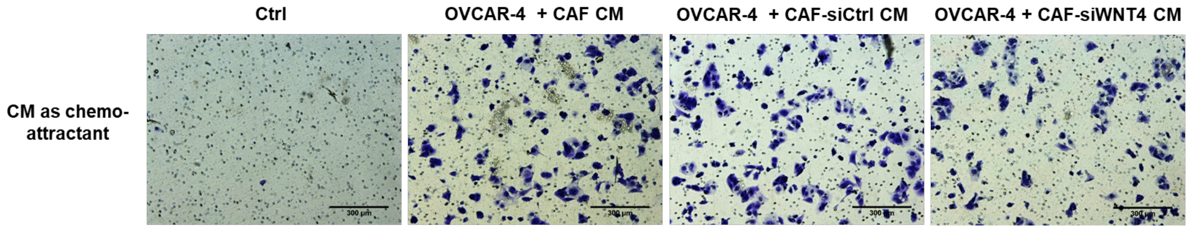

FIGURE S8

Representative microscopic images of migrated OVCAR4 cells in response to CM from untransfected CAF (CAF CM), from control transfected CAF (CAF siCtrl CM) or from siRNA-WNT4 transfected CAF (CAF siWNT4 CM) used as chemoattractant. A background control (Ctrl) of migrating tumor cells in the absence of any chemoattractant is including (left panel).

A

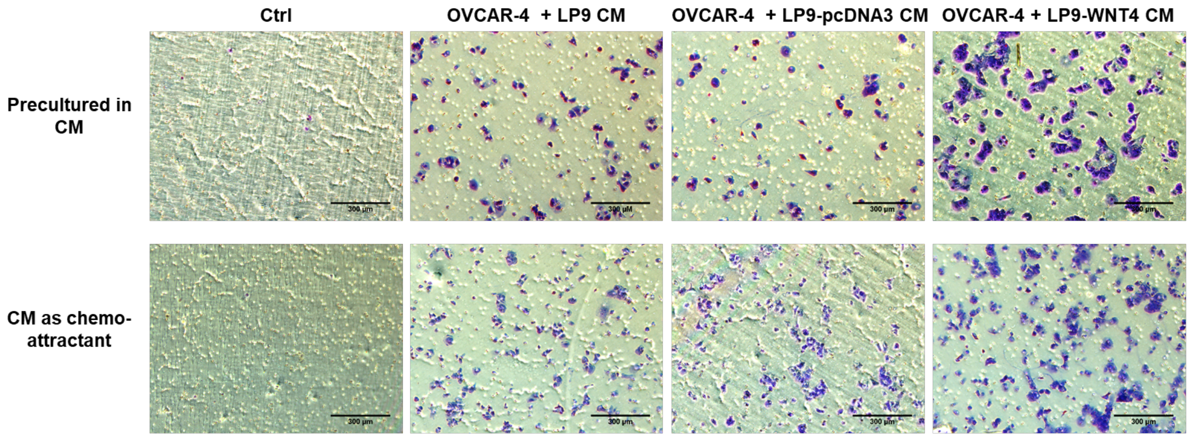

B

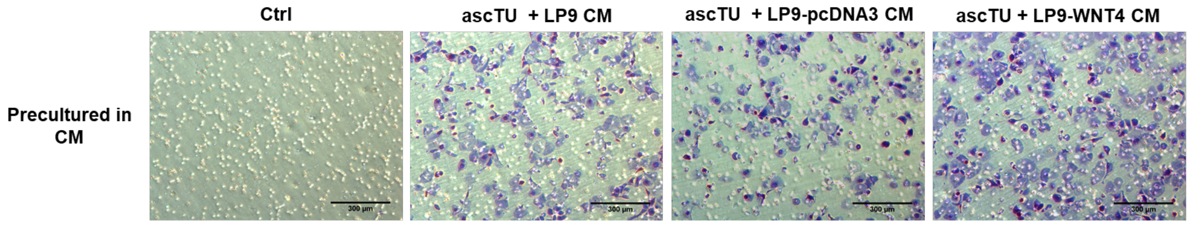

FIGURE S9

Representative microscopic images of migrated OVCAR4 cells (A) and primary ascites-derived HGSC cells (ascTU) (B) in response to WNT4. CM from WNT4-overexpressing, from control-transfected (pcDNA3) or from untransfected LP9 cells were either used as chemoattractant (A) or for preincubation of tumor cells prior to migration towards 10% FCS as the chemoattractant (A and B). A background control (Ctrl) of migrating tumor cells in the absence of any chemoattractant is including (left panel).

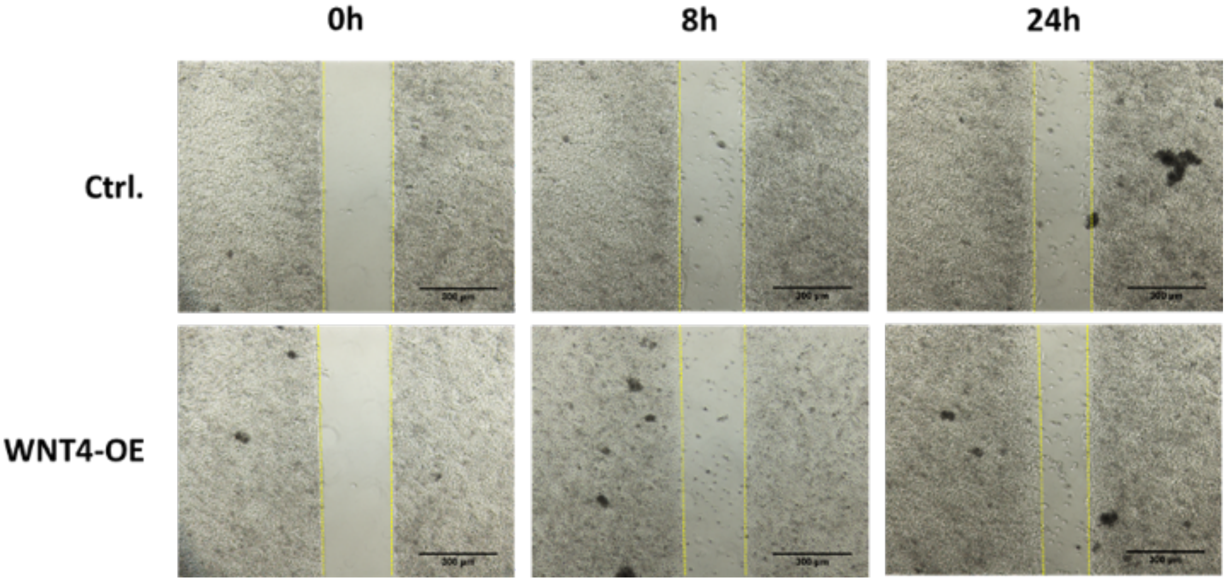

FIGURE S10
Representative microscopic images of wound closure in OVCAR4 monolayers (scratch assay) stimulated by CM of WNT4-overexpressing LP9 cells (WNT-OE) after 0, 8 and 24 hrs. Quantification of wound closure are described in Fig. 5D (n=5).

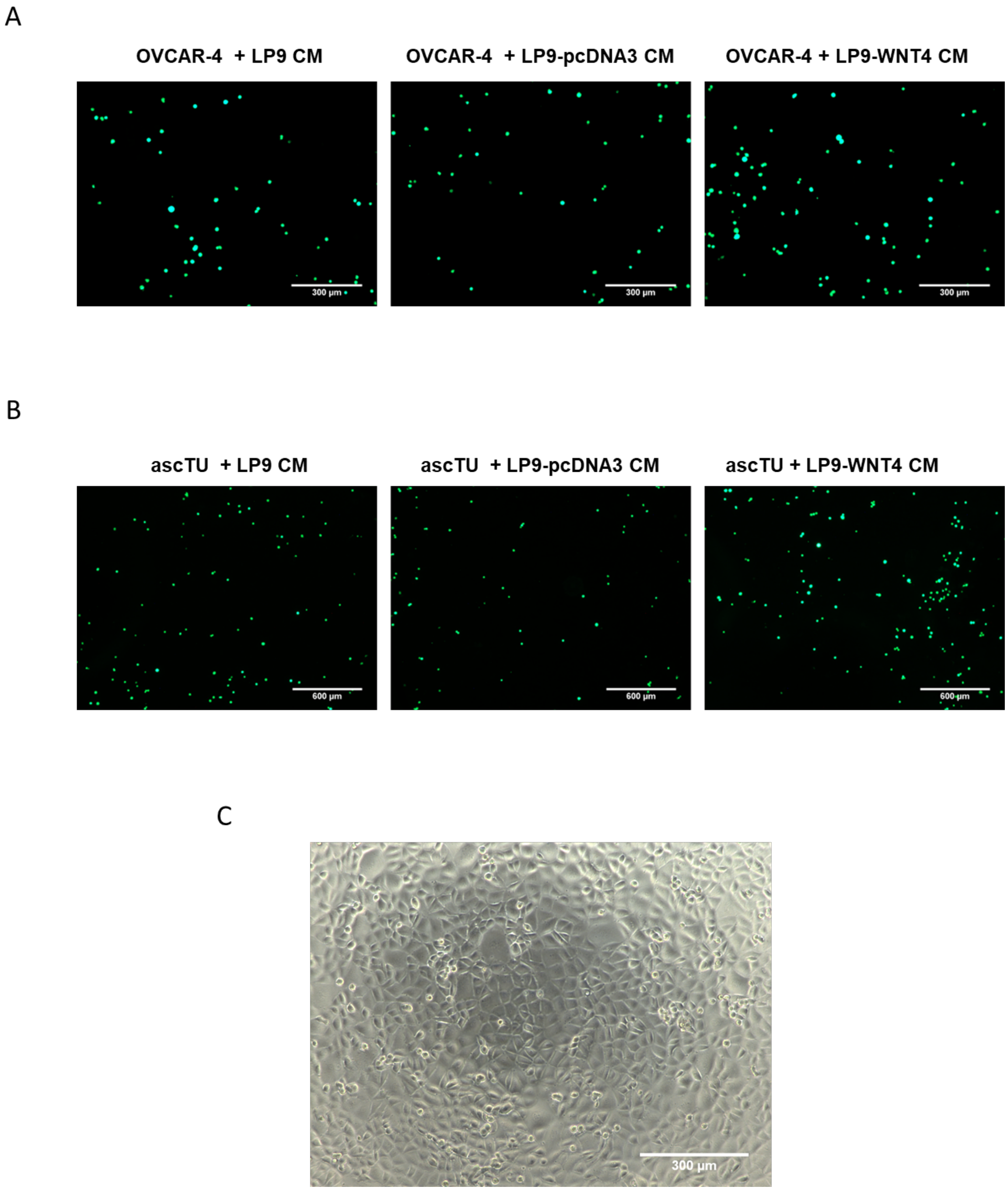

FIGURE S11

Representative images of tumor cell adhesion to a confluent monolayer of peritoneal mesothelial cells (MESO) after stimulation of tumor cells with CM of WNT4-overexpressing, control-transfected (pcDNA3) or untransfected LP9 cells. Adhesion of CellTracker green-labeled OVCAR4 cells (A) and ascites-derived primary HGSC cells (ascTU) (B) is shown after 1h (OVCAR4) or 2h (ascTU) co-culture on a MESO monolayer. (C) Microscopic evaluation of the integrity of the MESO monolayer used for adhesion assays (well of 96-well plate).

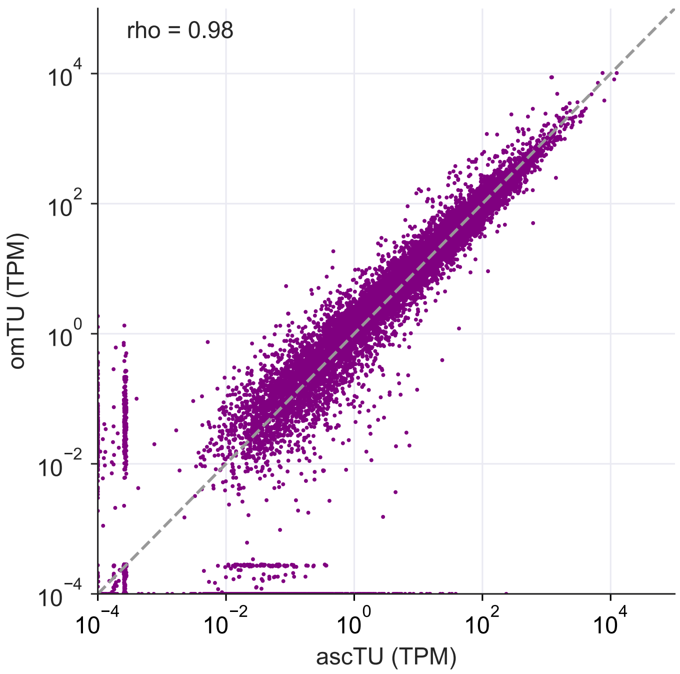

FIGURE S12

Scatter plot showing the correlation of all genes expressed in omTU versus ascTU based on the RNA-Seq data in Table S7 (medians).

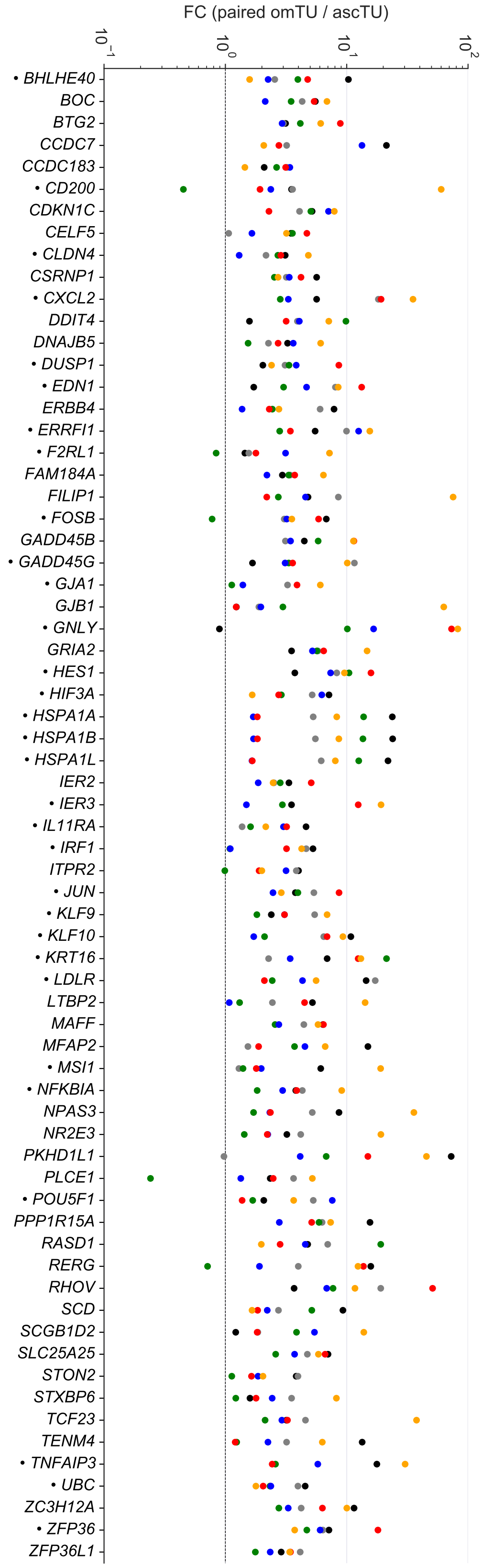

FIGURE S13

Genes upregulated in omTU versus ascTU (RNA-Seq; Table S14) and associated with the term "pro-inflammatory" in the genecards.org database. Genes also associated with "epithelial differentiation" in the genecards.org database are marked by dots.

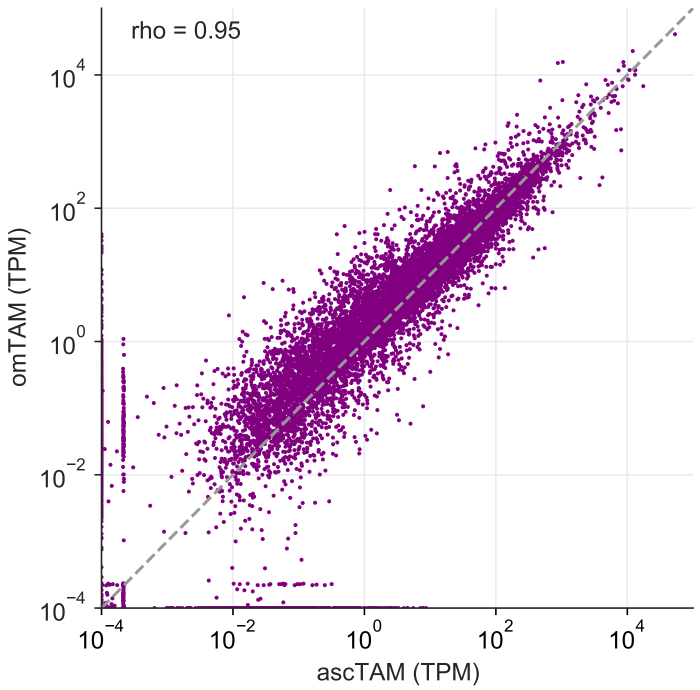

FIGURE S14
Scatter plot showing the correlation of all genes expressed in omTAM versus ascTAM based on the RNA-Seq data in Table S7 (medians).

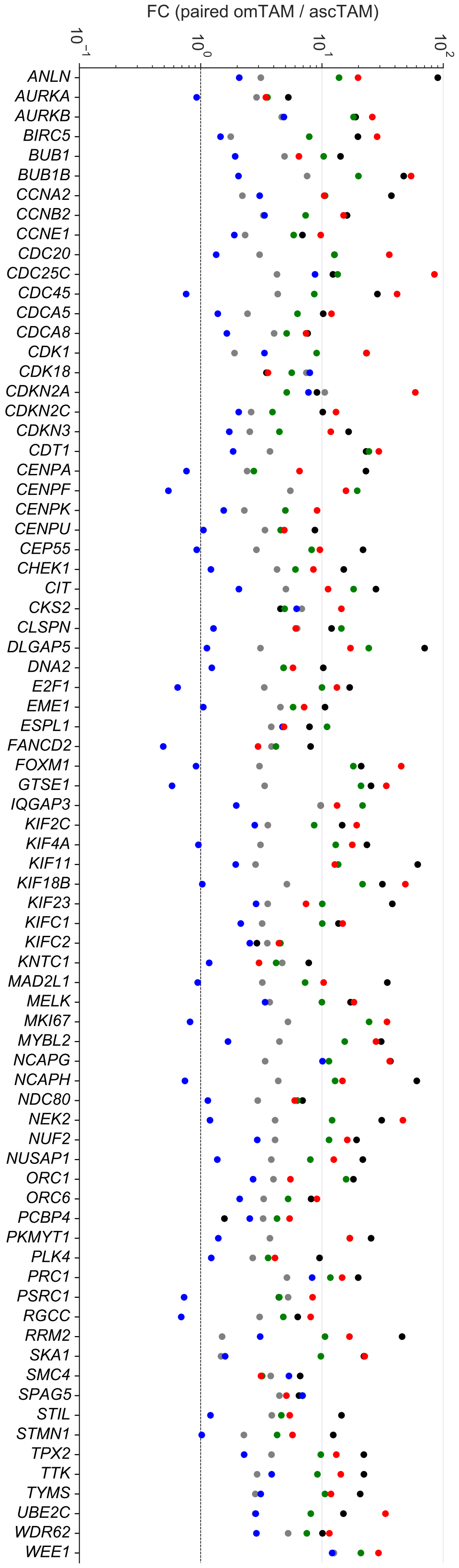

FIGURE S15

Genes upregulated in omTAM versus ascTAM (RNA-Seq; Table S15) and associated with the GO term "mitotic cell cycle process".

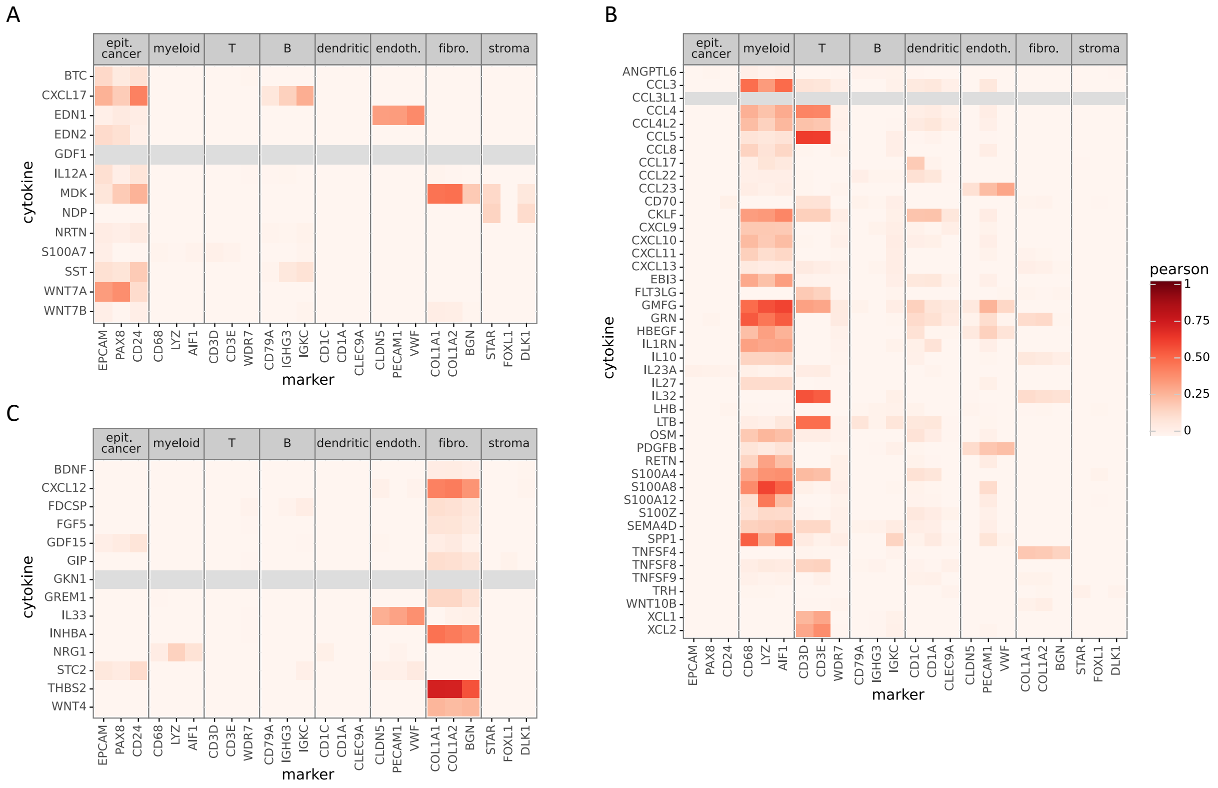

FIGURE S16

Pearson correlation analysis (on single cell gene expression) of cytokine genes expressed by omTU, immune cells or omental CAF in the present study with cell-type-selective marker genes in a published scRNA-Seq dataset for ovarian cancer.

Single cell count matrix data from Olbrecht et al. (2021) were processed as described by the authors, and Pearson correlation between cytokine genes identified as cell type specific (present study, Fig. 3) and cell type marker genes from Olbrecht et al. (2021) were calculated. Pearson correlation smaller than 0 was clipped to 0. Grey entries signify genes not present in scRNA-Seq dataset.

(A) Tumor cell specific cytokine group.
(B) Immune specific cytokine group.
(C) CAF specific cytokine group.

epit. cancer: epithelial cancer; endoth: endothelial; fibro.: fibroblast; stroma: normal ovarian stroma.

Reference:
Olbrecht, S, Busschaert, P, et al. High-grade serous tubo-ovarian cancer refined with single-cell RNA sequencing: specific cell subtypes influence survival and determine molecular subtype classification. *Genome Med* 2021; 13:111.
